# Subcortical association with memory performance in schizophrenia: a structural magnetic resonance imaging study

**DOI:** 10.1038/s41398-017-0069-3

**Published:** 2018-01-10

**Authors:** Daisuke Koshiyama, Masaki Fukunaga, Naohiro Okada, Fumio Yamashita, Hidenaga Yamamori, Yuka Yasuda, Michiko Fujimoto, Kazutaka Ohi, Haruo Fujino, Yoshiyuki Watanabe, Kiyoto Kasai, Ryota Hashimoto

**Affiliations:** 1 Department of Neuropsychiatry, Graduate School of Medicine, The University of Tokyo, Tokyo, Japan; 2 Division of Cerebral Integration, National Institute for Physiological Sciences, Aichi, Japan; 3 Division of Ultrahigh Field MRI, Institute for Biomedical Sciences, Iwate Medical University, Iwate, Japan; 4 Department of Psychiatry, Osaka University Graduate School of Medicine, Osaka, Japan; 5 Oncology Center, Osaka University Hospital, Osaka, Japan; 6 Graduate School of Human Sciences, Osaka University, Osaka, Japan; 7 Diagnostic and Interventional Radiology, Osaka University Graduate School of Medicine, Osaka, Japan; 8 Molecular Research Center for Children’s Mental Development, United Graduate School of Child Development, Osaka University, Osaka, Japan

## Abstract

Memory performance is severely impaired in individuals with schizophrenia. Although several studies have reported a relationship between memory performance and hippocampal volume, only a few structural magnetic resonance imaging (MRI) studies have investigated the relationship between memory performance and subcortical structures other than hippocampus in patients with schizophrenia. We investigated the relationship between memory performance and subcortical regional volumes in a large sample of patients with schizophrenia. Participants included 174 patients with schizophrenia and 638 healthy comparison subjects (HCS). The Wechsler Memory Scale-Revised (WMS-R) has three memory indices (verbal immediate recall, visual immediate recall, and delayed recall (verbal plus visual)) and one control neurocognitive index (attention/concentration). We obtained T1-weighted MRI data and measured the bilateral volumes of the hippocampus, amygdala, thalamus, nucleus accumbens (NA), caudate, putamen, and globus pallidus. Patients with schizophrenia had significantly lower scores for all of the indices of the WMS-R than the HCS. They had more severe impairments in verbal immediate recall and delayed recall than in visual immediate recall and attention/concentration. Verbal immediate recall/delayed recall scores in patients with schizophrenia were significantly correlated not only with hippocampal volume (left: *r* = 0.34; right: *r* = 0.28/left: *r* = 0.33; right: *r* = 0.31), but also with NA volume (left: *r* = 0.24; right: *r* = 0.25/left: *r* = 0.26; right: *r* = 0.27). The present investigation with a large sample size did not only replicate hippocampal volume and memory association, but also found that NA volume is associated with memory performances in schizophrenia.

## Introduction

Cognitive dysfunction is a core feature of schizophrenia, which seriously affects every day social functioning. Among cognitive domains, memory is markedly impaired in patients with schizophrenia when compared to healthy comparison subjects (HCS)^1–3^. In particular, verbal memory has been identified as a predictor of functional outcome in schizophrenia^4,5^. However, improving cognitive dysfunction, including memory impairments, in schizophrenia has been a challenge. The effects of available pharmacotherapy and cognitive training approaches are limited with regard to improving cognitive dysfunction in schizophrenia^6–9^. A better understanding of the biological bases of memory is important in developing evidence-based biological and psychosocial intervention strategies for the treatment of memory impairment, which significantly affects daily life in individuals with schizophrenia.

Correlational analysis of magnetic resonance imaging (MRI) volumes and performance indices has been shown to be a reliable strategy for investigating the bases of cognitive and behavioral deficits in schizophrenia^10^. Previous studies have found a robust association between memory performance and prefrontal and hippocampal volume in patients with schizophrenia^11–14^. In contrast, only a few studies have investigated the association between memory performance and MRI volumes of subcortical regions of interest. These limited number of studies have shown a significant negative correlation between putamen/amygdala volume and verbal memory in patients with schizophrenia^15,16^, and shed light on the function of subcortical structures on memory performance in patients with schizophrenia. These studies suggest that subcortical structures other than hippocampus may also have important role on memory performance; thus, further study is needed to show the relationships of subcortical structures with memory performance in patients with schizophrenia.

The purpose of the present study was to explore the relationship between memory performance and subcortical volumes in a large sample of patients with schizophrenia. We recently conducted a multicenter meta-analytic MRI study of limbic and subcortical regions of interest in patients with schizophrenia and found altered subcortical regional volumes in schizophrenia^17^. The current investigation is a subsample analysis of the associations of memory performance.

## Methods

### Subjects

The participants included 174 patients with schizophrenia and 638 HCS (Table 1). They had already participated in separate previous studies of cognitive function and neuroimaging^17–19^. Participants with schizophrenia were recruited from outpatient and inpatient units at Osaka University Hospital. Each patient with schizophrenia was diagnosed by at least two trained psychiatrists according to the criteria provided in the Diagnostic and Statistical Manual of Mental Disorders, Fourth Edition (DSM-IV) based on a Structured Clinical Interview for DSM-IV (SCID). The HCS were recruited through local advertisements at Osaka University. The HCS were evaluated using the non-patient version of the SCID to exclude individuals who had current or past contact with psychiatric services or who had received psychiatric medication. The subjects were excluded if they had neurological or medical conditions that could potentially affect the central nervous system, such as atypical headache, head trauma with loss of consciousness, chronic lung disease, kidney disease, chronic hepatic disease, thyroid disease, active cancer, cerebrovascular disease, epilepsy, seizures, substance-related disorders, or mental retardation.

**Table 1:** Demographic and clinical characteristics of the participants

	SZ (*N* = 174)		HCS (*N* = 638)		Statistics		
	Mean	SD	Mean	SD	Effect size, *d*	*t* or *χ*^2^	*p*
Age	34.5	11.6	33.9	12.9	0.05	*t* (810) = −0.56	0.57
Male/female	98/76		315/323			*χ*^2^ (1.0) = 2.64	0.10
Education (years)	13.9	2.6	15.0	2.1	−0.46	*t* (810) = 5.78	1.1 × 10^−8^
Premorbid IQ^a^	102.0	10.1	108.4	7.7	−0.71	*t* (806) = 8.93	3.0 × 10^−18^
Duration of illness (years)	10.9	8.9					
PANSS positive^b^	18.9	5.7					
PANSS negative^b^	20.0	5.6					
PANSS general^b^	43.3	10.7					
PANSS total^b^	82.1	20.0					
*Medication*							
Chlorpromazine equivalent (mg/day)	581.7	519.8					
Diazepam equivalent (mg/day)	8.5	28.7					
Biperiden equivalent (mg/day)	0.9	1.7					

Underline indicates *p* < 0.05

*SZ* patients with schizophrenia, *HCS* healthy comparison subjects, *SD* standard deviation, *IQ* intelligence quotient, *PANSS* positive and negative syndrome scale

^a^*N* = 170; four patients with schizophrenia have no premorbid IQ data, ^b^*N* = 173; one patient with schizophrenia has no PANSS data

Estimated premorbid intelligence quotient (IQ) was assessed using the Japanese version of the National Adult Reading Test^20^. Psychotic symptoms were evaluated using the Positive and Negative Syndrome Scale^21^. Doses of antipsychotics, anxiolytics/sedatives/hypnotics and antiparkinsonian drugs received by the patients were converted to chlorpromazine, diazepam, and biperiden equivalents, respectively^22^. Written informed consent was obtained from each subject before participation. This study was approved by the Research Ethical Committee of Osaka University and the Ethical Committee of the Faculty of Medicine, the University of Tokyo.

### Wechsler Memory Scale-revised indices

Memory performance was measured using the Wechsler Memory Scale-Revised (WMS-R)^23^. The WMS-R has three memory indices and one control general cognitive index (attention/concentration). The Verbal Memory Index and the Visual Memory Index are measures of immediate recall. The Delayed Recall Index consists of verbal and visual delayed recall scores. The raw scores of the WMS-R were converted to scaled scores to normalize for age. The mean time interval between the WMS-R measurements and the MRI scanning was 35.7 days (SD, 133.6 (days); range, 0–1473 (days)).

### Image analysis

We performed MRI and obtained T1-weighted images using two machines: Osaka A and Osaka B. Overall, 107 patients with schizophrenia and 401 HCS were scanned using Osaka A, and 67 patients and 237 HCS were scanned using Osaka B. For Osaka A, the scanner type was a GE 1.5 T, Signa EXCITE. T1-weighted images, using a fast spoiled gradient echo (SPGR) and a head QD coil, were acquired with the following parameters: repetition time (TR) = 12.6 ms, echo time (TE) = 4.2 ms, inversion time (TI) = 400 ms, flip angle = 15 degrees, matrix = 256 × 256 × 124, field of view (FOV) = 240 × 240 × 172 mm, voxel size = 0.9375 × 0.9375 × 1.4 mm, slice thickness = 1.4 mm, and number of slices = 124. The slices were oriented in the sagittal plane. For Osaka B, the scanner type was a GE 3.0 T, Signa HDxt. T1-weighted images, using a fast SPGR and 8HRBRAIN coil, were acquired with the following parameters: TR = 7.2 ms, TE = 2.9 ms, TI = 400 ms, flip angle = 11 degrees, matrix = 256 × 256 × 172, FOV = 240 × 240 × 172 mm, voxel size = 0.9375 × 0.9375 × 1 mm, slice thickness = 1 mm, number of slices = 172. The slices were oriented in the sagittal plane.

Image processing was performed as previously described in a previous study by our group authored by Okada et al.^17^ Two independent researchers visually checked original T1-weighted images for quality control. Images with partial deficits (i.e., coverage problems), those with a low signal-to-noise ratio or artifacts (e.g., motion artifacts and magnetic susceptibility artifacts), and those with organic abnormal findings (e.g., large cerebellar cyst and cavum septum pellucidum) were excluded. Next, T1-weighted imaging data that had passed the first quality-control step were processed using FreeSurfer software version 5.3 (http://surfer.nmr.mgh.harvard.edu)^24^. Through this procedure, we obtained images of subcortical segmentation and regional volumes (for bilateral hippocampus, amygdala, thalamus, nucleus accumbens (NA), caudate, putamen, globus pallidus, and the intracranial volume (ICV)). Two independent researchers visually inspected each segmentation image in order to exclude images with poor parcellation. No subject was excluded because of FreeSurfer processing failure. After the two quality-control steps (inspection of original MRI data and screening for successful FreeSurfer processing), we obtained raw subcortical volume data. Our analysis follows the methods used in the study by van Erp et al. from the Enhancing Neuro Imaging Genetics through Meta-Analysis Schizophrenia Working Group (ENIGMA-SZ)^25^. We used corrected regional volumes to remove the effects of confounding factors. A corrected volume was defined as follows. We first performed a linear regression using the following formula:

$$\begin{array}{rcl}{\mathrm{Raw}}\;{\mathrm{volume}} & = & {\mathrm{\beta }}_{\mathrm{1}} \times {\mathrm{age}} + {\mathrm{\beta }}_{\mathrm{2}} \times {\mathrm{sex}} \\&&+ {\mathrm{\beta }}_{\mathrm{3}} \times {\mathrm{ICV}} + {\mathrm{\beta }}_{\mathrm{4}} \times {\mathrm{machine}} + {\mathrm{\varepsilon }}{\mathrm{.}}\end{array}$$

Dummy variables were created for sex (male = 1, female = 2) and for the machine (Osaka A = 1, Osaka B = 2). *ε* means the corrected volume. We used corrected regional volumes (bilateral hippocampus, amygdala, thalamus, NA, caudate, putamen, and globus pallidus) as MRI indices in our statistical analysis.

Because the NA is a relatively small structure^25^, we performed manual tracing of NA by three blinded raters on 40 randomly selected subjects (10 with schizophrenia in Osaka A, 10 with schizophrenia in Osaka B, 10 with HCS in Osaka A, and 10 with HCS in Osaka B) for confirmation purpose of segmentation of NA with FreeSurfer. The methods and results are shown in Supplementary Text, Supplementary Table 1–4, and Supplementary Figure 1.

### Statistical analysis

All statistical analyses were conducted using SPSS version 23.0.0.0 (IBM Corp., Armonk, NY). For comparisons of demographic data, clinical characteristics, and neuropsychological measures between patients and HCS, we used a significance threshold of *p* < 0.05 for *t*-tests and *χ*^2^-tests. For each demographic or neuropsychological measure, Cohen’s *d* effect sizes were calculated from the overall group contrast. Histograms of the age distribution in both groups are shown in Supplementary Figure 2.

We performed a repeated-measures analysis of variance (ANOVA) with diagnostic groups (schizophrenia and HCS) as the between-subjects factors and the four WMS-R (three memory and one general cognitive) indices as the within-subject factors. If the group-by-index interaction was significant, we then performed post hoc repeated-measures ANOVAs with diagnostic group as the between-subjects factor and the WMS-R index as the within-subject factor, for each of the combinations of pairs of indices (six combinations). We performed these analyses to investigate which combination of indices was responsible for the interaction. We used Bonferroni correction (*p* value < 8.3 × 10^−3^ (0.05/6) was considered statistically significant). Results of the Greenhouse–Geisser correction were reported when Mauchly’s test for sphericity was significant.

For comparisons of the MRI indices between patients and HCS, we used a significance threshold of *p* < 0.05 for *t*-tests. Here we did not apply correction for multiple comparisons, as our previous larger-scale meta-analysis study of subcortical volume abnormalities in schizophrenia^17^ included the current sample. Thus, the purpose of this volume comparison was only to confirm the previous findings in this subsample. For each MRI index, Cohen’s *d* effect sizes were calculated from the overall group contrast. Furthermore, we showed the Pearson correlation coefficients (*r*) map among regional brain volumes to show the relationships both within and across groups, respectively.

To investigate the relationship between memory performance and the subcortical volumes, we separately calculated Pearson’s correlations coefficient (*r*) between the three memory indices of Verbal Memory, Visual Memory, and Delayed Memory, and the MRI indices in patients with schizophrenia and HCS. Correlation models were examined for each of the 14 anatomical regions and the three memory indices, and a *p* value of <1.19 × 10^−3^ (0.05/42 (three memory indices × 14 regions)) was considered statistically significant based on the Bonferroni correction. We conducted a power analysis to estimate the sample sizes required to detect an effect size with previous study^14^ at a power of 0.95 and a one-tailed significance level of 1.19 × 10^−3^ (0.05/42), with G*Power version 3.1.9.2^26^.

If we found a significant correlation between a certain memory index and a MRI index in the schizophrenia group, we further tested whether the correlation was specific to schizophrenia by using Fisher’s *r* to *z* transformation (one-tailed) to compare the correlations between patients with schizophrenia and HCS. A *p* value of <0.05, with Bonferroni correction, utilized for correction during multiple comparisons, was considered statistically significant.

We also tested whether correlation with an MRI index was specific to memory or whether it was nonspecific and associated with general cognitive performance (attention/concentration index). First, we computed the Pearson’s correlation coefficient between the Attention/Concentration index and the MRI index in patients with schizophrenia. The significance level was set at a *p* value of <3.57 × 10^−3^ (0.05/14) based on Bonferroni correction. Then, we calculated the differences in coefficients between the memory MRI and general cognitive MRI correlations using Fisher’s *r* to *z* transformation (one-tailed) to compare the coefficients between the memory MRI and general cognitive MRI correlations. A *p* value of <0.05, with Bonferroni correction, utilized for correction during multiple comparisons, was considered statistically significant. Furthermore, we performed partial correlation analyses between Verbal Memory/Delayed Recall and NA volumes to adjust for Attention/Concentration in the schizophrenia group. A *p* value of <0.05 was considered statistically significant. Here, we did not use Bonferroni correction because we sought to pick up potential confounders in a sensitive manner.

We performed partial correlation analyses between the verbal immediate recall/delayed recall and the NA volumes adjusting for hippocampal volumes to investigate whether the NA volumes had unique associations with memory performance in patients with schizophrenia. Potential effects of the premorbid IQ on the memory performance were examined with correlation analyses between premorbid IQ and the three memory scores in the schizophrenia group. Furthermore, we performed partial correlation analyses between verbal immediate recall/delayed recall and hippocampus/NA volume adjusting for premorbid IQ in patients with schizophrenia. Potential effects of the duration of illness on the hippocampus/NA volume were tested with correlation analyses between the duration of illness and the hippocampus/NA volume in patients. In addition, we performed partial correlation analyses between the verbal immediate recall/delayed recall and hippocampus/NA volume adjusting for duration of illness in patients. We evaluated effects of time interval between the WMS-R measurements and the MRI scanning on our main finding of associations between verbal immediate recall/delayed recall and hippocampus/NA volume with partial correlation analyses. Furthermore, we performed correlation analyses between verbal immediate recall/delayed recall and hippocampus/NA volume only in 147 patients with schizophrenia within 3 months' time interval between the WMS-R measurements and the MRI scanning.

In this study, we used two MRI scanners of different strengths. Statistical corrections to account for the difference may be insufficient to accurately capture the differences between MRI scanners. Therefore, to confirm the credibility of our data, we performed correlation analyses on our main findings in the schizophrenia group, for each MRI scanner: Osaka A and Osaka B. We used regional volumes that were corrected by regressing the effects of age, sex, and ICV. A *p* value of <0.05 was considered statistically significant.

Finally, we sought to partial out the medication effect on our main finding of associations between verbal immediate recall/delayed recall and hippocampus/NA volume in the schizophrenia group. We first calculated Pearson’s *r* between medication dosage (chlorpromazine, diazepam, and biperiden equivalents) and those memory and MRI indices. Bonferroni correction was not used here because we sought to pick up potential confounders in a sensitive manner. If there was a significant correlation with medication dosage, the partial correlation with adjustments for medication was calculated for each of the combinations of the WMS-R and MRI indices. A *p* value of <0.05 was considered statistically significant.

## Results

### WMS-R indices

We performed a repeated-measures ANOVA to show which memory index is more impaired in schizophrenia than in HCS. A repeated-measures ANOVA indicated a significant group-by-index interaction (Greenhouse–Geisser corrected: F_2.23, 1805.93_ = 54.32, *p* = 5.9 × 10^−26^). Post hoc repeated-measures ANOVAs with diagnostic groups as the between-subjects factors and the WMS-R indices as the within-subject factors revealed significant group-by-index interactions (Verbal Memory Index vs. Visual Memory Index: F_1, 810_ = 47.07, *p* = 1.4 × 10^−11^; Verbal Memory Index vs. Attention/Concentration Index: F_1, 810_ = 57.29, *p* = 1.0 × 10^−13^; Delayed Recall Index vs. Visual Memory Index: F_1, 810_ = 107.98, *p* = 7.9 × 10^−24^; Delayed Recall Index vs. Attention/Concentration Index: F_1, 810_ = 86.05, *p* = 1.6 × 10^−19^). However, the group-by-index interaction was not significant when the two indices of the WMS-R were Verbal Memory Index and Delayed Recall Index (F_1, 810_ = 4.84, *p* = 2.8 × 10^−2^), and Visual Memory Index and Attention/Concentration Index (F_1, 810_ = 3.23, *p* = 7.3 × 10^−2^). These findings indicate that verbal immediate recall and delayed recall are more severely affected than visual immediate recall and attention/concentration in patients with schizophrenia.

**Fig. 1:**
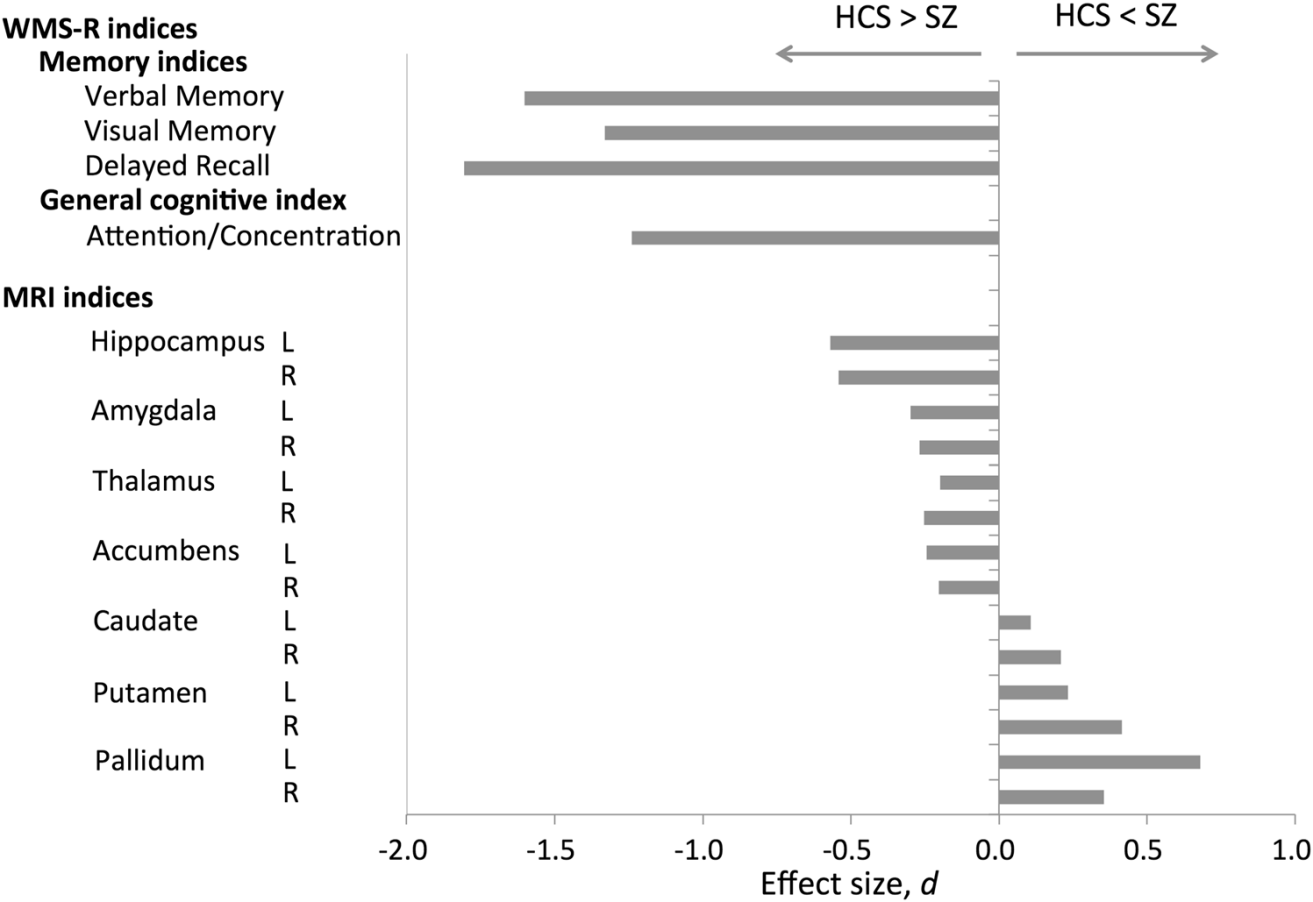
Cohen’s *d* effect sizes for differences in the WMS-R and MRI indices between patients with schizophrenia and HCS. The vertical axis represents the WMS-R and MRI indices. The horizontal axis represents Cohen’s *d* effect sizes for difference in the WMS-R and MRI indices between patients with schizophrenia and HCS. *WMS-R* Wechsler Memory Scale-Revised, *MRI* magnetic resonance imaging, *L* left, *R* right, *SZ* patients with schizophrenia group, *HCS* healthy comparison subjects group

All four indices of the WMS-R were significantly lower in patients with schizophrenia than in HCS with *t*-tests (Verbal Memory Index, *d* = −1.60, *p* = 2.0 × 10^−79^; Visual Memory Index, *d* = −1.33, *p* = 4.8 × 10^−67^; Delayed Recall Index, *d* = −1.81, *p* = 1.4 × 10^−101^; Attention/Concentration Index, *d* = −1.24, *p* = 8.5 × 10^−47^; Table 2, Fig. 1).

**Table 2:** Comparison of the WMS-R and MRI indices between patients with schizophrenia and HCS

		SZ (*N* = 174)		HCS (*N* = 638)		Statistics		
		Mean	SD	Mean	SD	Effect size, *d*	*t*	*p*
*WMS-R indices*								
Memory indices								
Verbal memory		83.6	20.9	112.2	14.0	−1.60	*t* (810) = 21.15	2.0 × 10^−79^
Visual memory		85.4	18.6	105.2	9.6	−1.33	*t* (810) = 19.03	4.8 × 10^−67^
Delayed recall		79.3	20.3	109.5	12.0	−1.81	*t* (810) = 24.82	1.4 × 10^−101^
General cognitive index								
Attention/concentration		92.6	15.3	110.1	12.8	−1.24	*t* (810) = 15.34	8.5 × 10^−47^
*MRI indices*								
Hippocampus	L	2922.1	412.9	3140.1	340.9	−0.58	*t* (810) = 7.14	2.0 × 10^−12^
	R	2386.6	407.6	2576.9	327.8	−0.51	*t* (810) = 6.43	2.2 × 10^−10^
Amygdala	L	872.1	154.0	913.2	161.6	−0.26	*t* (810) = 3.01	2.7 × 10^−3^
	R	1142.0	200.2	1186.4	187.8	−0.23	*t* (810) = 2.73	6.6 × 10^−3^
Thalamus	L	3119.3	708.5	3299.8	673.2	−0.26	*t* (810) = 3.11	2.0 × 10^−3^
	R	2605.9	602.7	2752.0	556.1	−0.25	*t* (810) = 3.04	2.5 × 10^−3^
Accumbens	L	851.2	98.1	873.3	96.9	−0.23	*t* (810) = 2.67	7.7 × 10^−3^
	R	647.5	93.6	665.8	83.3	−0.21	*t* (810) = 2.49	1.3 × 10^−2^
Caudate	L	1799.1	361.2	1760.3	385.3	0.10	*t* (810) = −1.18	0.24
	R	2371.2	380.6	2291.9	403.3	0.20	*t* (810) = −2.30	2.2 × 10^−2^
Putamen	L	5914.8	578.1	5777.2	577.1	0.24	*t* (810) = −2.79	5.4 × 10 ^−3^
	R	5958.9	522.0	5761.8	507.9	0.38	*t* (810) = −4.51	7.6 × 10 ^−6^
Pallidum	L	1407.3	224.3	1255.0	202.2	0.71	*t* (810) = −8.60	4.0 × 10 ^−17^
	R	1212.6	171.5	1147.1	173.1	0.38	*t* (810) = −4.42	1.1 × 10 ^−5^

The unit for the MRI indices is mm^3^. Underline indicates *p* < 0.05

*WMS-R* Wechsler Memory Scale-Revised, *MRI* magnetic resonance imaging, *SZ* patients with schizophrenia, *HCS* healthy comparison subjects, *SD* standard deviation, *L* left, *R* right

### MRI indices

The corrected regional volumes of bilateral hippocampus (left: *d* = −0.58, *p* = 2.0 × 10^−12^; right: *d* = −0.51, *p* = 2.2 × 10^−10^), amygdala (left: *d* = −0.26, *p* = 2.7 × 10^−3^; right: *d* = −0.23, *p* = 6.6 × 10^−3^), thalamus (left: *d* = −0.26, *p* = 2.0 × 10^−3^; right: *d* = −0.25, *p* = 2.5 × 10^−3^), and NA (left: *d* = −0.23, *p* = 7.7 × 10^−3^; right: *d* = −0.21, *p* = 1.3 × 10^−2^) were significantly smaller in patients with schizophrenia than in HCS (Table 2, Fig. 1). On the other hand, the corrected regional volumes of the right caudate (*d* = 0.20, *p* = 2.2 × 10^−2^, bilateral putamen (left: *d* = 0.24, *p* = 5.4 × 10^−3^; right: *d* = 0.38, *p* = 7.6 × 10^−6^), and bilateral pallidum (left: *d* = 0.71, *p* = 4.0 × 10^−17^; right: *d* = 0.38, *p* = 1.1 × 10^−5^) were significantly larger in patients with schizophrenia than in HCS (Table 2, Fig. 1). The correlation maps among the regional brain volumes are shown in Supplementary Figure 3 for all participants, Supplementary Figure 4 for schizophrenia group, and Supplementary Figure 5 for HCS.

### Correlation analyses

**Fig. 2:**
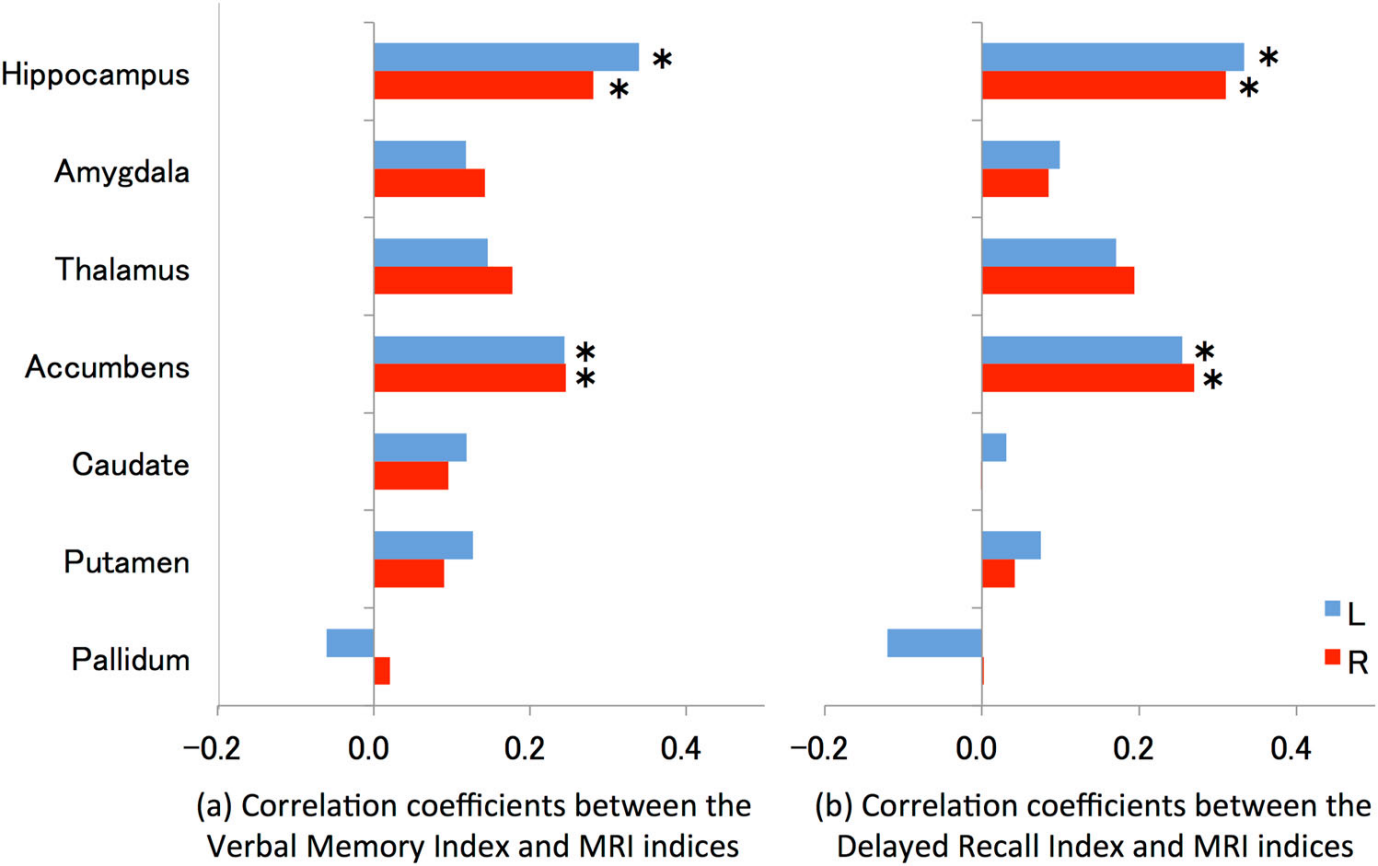
Correlation coefficients between Verbal Memory/Delayed Recall indices and MRI indices in patients with schizophrenia. Correlation between (**a**) the Verbal Memory Index and MRI indices and (**b**) the Delayed Memory Index and MRI indices. The vertical axis represents MRI indices. The horizontal axis represents the Pearson correlations coefficient *r* between the Verbal Memory/Delayed Recall indices and MRI indices. The asterisk indicates a significant correlation between the Verbal Memory/Delayed Recall indices and MRI indices. *MRI* magnetic resonance imaging, *L* left, *R* right

In patients with schizophrenia, the Verbal Memory (verbal immediate recall) Index was significantly correlated with the bilateral volumes of the hippocampus (left: *r* = 0.339, *p* = 4.6 × 10^−6^; right: *r* = 0.281, *p* = 1.8 × 10^−4^) and NA (left: *r* = 0.244, *p* = 1.17 × 10^−3^; right: *r* = 0.246, *p* = 1.1 × 10^−3^; Table 3, Fig. 2, Supplementary Figure 6, 7). The Visual Memory (visual immediate recall) Index was significantly associated with bilateral hippocampal volumes (left: *r* = 0.361, *p* = 9.8 × 10^−7^; right: *r* = 0.410, *p* = 2.0 × 10^−8^) and the right thalamic volume (*r* = 0.270, *p* = 3.2 × 10^−4^; Table 3, Fig. 2). The Delayed Recall Index was also significantly correlated with the bilateral volumes of the hippocampus (left: *r* = 0.333, *p* = 6.9 × 10^−6^; right: *r* = 0.310, *p* = 3.2 × 10^−5^) and the NA (left: *r* = 0.255, *p* = 7.0 × 10^−4^; right: *r* = 0.270, *p* = 3.1 × 10^−4^; Table 3, Fig. 2, Supplementary Figure 6, 7). We found no significant correlations between any of the memory indices and the MRI indices in HCS (Table 3, Supplementary Figure 8, 9).

**Table 3:** Correlations between the WMS-R indices and MRI indices in patients with schizophrenia and healthy comparison subjects

		Hippocampus		Amygdala		Thalamus		Accumbens		Caudate		Putamen		Pallidum	
		L	R	L	R	L	R	L	R	L	R	L	R	L	R
*Patients with schizophrenia*															
Memory indices															
Verbal memory	*r*	0.339	0.281	0.118	0.142	0.145	0.177	0.244	0.246	0.118	0.095	0.127	0.089	−0.060	0.020
	*p*	4.6 × 10 ^−6^	1.8 × 10 ^−4^	6.1 × 10^−2^	5.5 × 10^−2^	5.5 × 10^−2^	1.9 × 10^−2^	1.17 × 10 ^−3^	1.1 × 10 ^−3^	0.12	0.21	0.10	0.24	0.43	0.79
Visual memory	*r*	0.361	0.410	0.196	0.079	0.236	0.270	0.170	0.192	0.033	0.005	0.039	0.013	−0.161	−0.012
	*p*	9.8 × 10 ^−7^	2.0 × 10 ^−8^	9.4 × 10^−3^	0.30	1.8 × 10^−3^	3.2 × 10 ^−4^	2.5 × 10^−2^	1.1 × 10^−2^	0.67	0.94	0.61	0.86	3.4 × 10^−2^	0.87
Delayed Recall	*r*	0.333	0.310	0.099	0.084	0.170	0.194	0.255	0.270	0.031	−0.002	0.075	0.041	−0.120	0.002
	*p*	6.9 × 10 ^−6^	3.2 × 10 ^−5^	0.19	0.27	2.5 × 10^−2^	1.1 × 10^−2^	7.0 × 10 ^−4^	3.1 × 10 ^−4^	0.68	0.98	0.33	0.59	0.11	0.97
General cognitive index															
Attention/Concentration	*r*	0.252	0.229	0.123	0.076	0.093	0.163	0.088	0.231	−0.013	−0.034	0.082	0.035	−0.033	0.080
	*p*	7.8 × 10 ^−4^	2.4 × 10 ^−3^	0.11	0.32	0.22	3.2 × 10^−2^	0.25	2.2 × 10 ^−3^	0.86	0.65	0.28	0.65	0.66	0.29
*Healthy comparison subjects*															
Memory indices															
Verbal memory	*r*	0.020	−0.024	0.004	−0.023	0.042	0.038	0.011	0.055	0.002	−0.015	−0.031	−0.042	−0.008	0.029
	*p*	0.61	0.55	0.91	0.56	0.29	0.34	0.79	0.16	0.95	0.70	0.44	0.29	0.84	0.46
Visual memory	*r*	−0.004	0.007	−0.008	0.021	−0.002	0.017	−0.030	0.003	−0.028	−0.038	0.024	−0.022	0.047	0.051
	*p*	0.93	0.87	0.84	0.60	0.96	0.67	0.45	0.93	0.48	0.34	0.55	0.58	0.24	0.20
Delayed recall	*r*	0.029	0.007	−0.003	0.024	0.058	0.067	0.034	0.067	0.027	0.012	−0.016	−0.015	0.022	0.059
	*p*	0.47	0.85	0.94	0.54	0.14	9.0 × 10^−2^	0.39	9.0 × 10^−2^	0.49	0.77	0.70	0.71	0.57	0.14
General cognitive index															
Attention/Concentration	*r*	−0.038	−0.042	0.009	−0.015	−0.039	−0.009	−0.005	−0.009	−0.016	0.023	0.034	0.028	<0.001	0.011
	*p*	0.34	0.28	0.82	0.71	0.32	0.81	0.89	0.83	0.68	0.56	0.39	0.49	1.00	0.78

Underline indicates *p* < 1.19 × 10^−3^ (0.05/42) for memory indices and *p* < 3.57 × 10^−3^ (0.05/14) for general cognitive index. *WMS-R* Wechsler Memory Scale-Revised, *MRI* magnetic resonance imaging, *L* left, *R* right, *r* Pearson correlation coefficient

Correlations between the Verbal Memory Index and NA volumes (left: *z* = 2.77, *p* = 2.8 × 10^−3^; right: *z* = 2.27, *p* = 1.2 × 10^−2^, one-tailed), and between the Delayed Recall Index and NA volumes (left: *z* = 2.63, *p* = 4.3 × 10^−3^; right: *z* = 2.44, *p* = 7.4 × 10^−3^, one-tailed) were specific to the schizophrenia group (the significance level was set at a *p* value of <1.3 × 10^−2^ (0.05/4) based on the Bonferroni correction).

Attention/Concentration was significantly correlated with the right NA volume in the schizophrenia group (*r* = 0.231, *p* = 2.2 × 10^−3^). The correlations between the Verbal Memory/Delayed Recall Index and left/right NA volumes were not stronger than the correlations between the Attention/Concentration Index and left/right NA volumes (Verbal Memory-Attention/Concentration, left: *z* = 1.49, *p* = 6.9 × 10^−2^; right: *z* = 0.15, *p* = 0.44; Delayed Recall-Attention/Concentration, left: *z* = 1.59, *p* = 5.6 × 10^−2^; right: *z* = 0.39, *p* = 0.35, one-tailed) in patients with schizophrenia. The significance level was set at a *p* value of < 1.3 × 10^−2^ (0.05/4) based on the Bonferroni correction. However, Verbal Memory/Delayed Recall was significantly correlated with NA volumes when adjusted for Attention/Concentration in the schizophrenia group (Supplementary Table 5).

The NA volumes were significantly correlated with Verbal Memory/Delayed Recall Index when adjusting for hippocampal volume except for the correlation between right NA and the Verbal Memory (but the correlation showed clear tendency, Supplementary Table 6). Because the premorbid IQ was significantly lower in the patients with schizophrenia, we performed correlational analyses to examine whether the premorbid IQ had an effect on the memory performance in the patients with schizophrenia. Premorbid IQ was significantly correlated with Verbal Memory (*r* = 0.464, *p* = 1.8 × 10^−10^), Visual Memory (*r* = 0.444, *p* = 9.6 × 10^−10^), and Delayed Recall Index (*r* = 0.444, *p* = 1.4 × 10^−9^) in the schizophrenia group. However, partial correlation analyses between verbal immediate recall/delayed recall and hippocampus/NA volume adjusting premorbid IQ showed almost the same results compared to the original results in the patients with schizophrenia (Supplementary Table 7).

We evaluated potential effects of the duration of illness on the hippocampus/NA volume with the correlation analyses between the duration of illness and the hippocampus/NA volume. The duration of illness was not significantly correlated with bilateral hippocampal volumes (left: *r* = 0.004, *p* = 0.96; right: *r* = −0.056, *p* = 0.46) or bilateral NA volumes (left: *r* = 0.009, *p* = 0.91; right: *r* = −0.046, *p* = 0.55) in patients with schizophrenia. In addition, partial correlation analyses between verbal immediate recall/delayed recall and hippocampus/NA volume adjusting duration of illness showed almost the same results compared to original results in the patients with schizophrenia (Supplementary Table 8).

We confirmed that time interval between the WMS-R measurements and the MRI scanning had no effect on our main findings of correlation between the Verbal Memory/Delayed Recall index and bilateral hippocampus/NA volumes with partial correlation analyses (Supplementary Table 9). We also performed correlation analyses between the Verbal Memory/Delayed Recall index and bilateral hippocampus/NA volumes only in 147 participants with schizophrenia within 3 months' time interval between the WMS-R measurements and the MRI scanning, and found almost same results compared to original results (Supplementary Table 9).

Upon conducting correlation analyses on our main findings in the schizophrenia group for each MRI machine, Osaka A and Osaka B, we could not replicate a significant correlation between Verbal Memory and bilateral NA volumes, or between Verbal Memory/Delayed Recall and the right hippocampal volume. However, a significant correlation between Delayed Recall and NA was replicated, and Verbal Memory/Delayed Recall was correlated with the right hippocampal volume at a trend level (Supplementary Table 10).

For the statistical considerations of the medication effect, the Verbal Memory/Delayed Recall index was significantly correlated with chlorpromazine equivalents; Delayed Recall index, bilateral hippocampal volumes, and right NA volume were significantly correlated with biperiden equivalents in the schizophrenia group. There were no significant correlations of diazepam equivalents with the Verbal Memory/Delayed Recall index or bilateral hippocampus/NA volumes in the schizophrenia group (Supplementary Table 11). We performed partial correlational analyses adjusted for chlorpromazine and biperiden equivalents between the Verbal Memory/Delayed Recall index and bilateral hippocampus/NA volumes in the schizophrenia group, and found that the all of correlations remained significant (Supplementary Table 12).

## Discussion

We observed that three memory indices and one general cognitive index of the WMS-R were significantly lower in patients with schizophrenia compared with HCS. Verbal immediate recall and delayed recall were more severely affected than visual immediate recall and attention/concentration in patients with schizophrenia. Verbal immediate recall and delayed recall were associated with the bilateral volumes of the hippocampus and the NA in patients with schizophrenia. In contrast, there were no significant correlations of verbal immediate recall/delayed recall with NA volume in HCS. Moreover, the correlation coefficient between memory indices and NA volume was statistically larger in patients with schizophrenia than in HCS. The correlation between verbal immediate recall/delayed recall and NA volume was not significantly stronger than the correlation between attention/concentration and NA volume. However, verbal immediate recall/delayed recall was significantly correlated with NA volume when adjusted for attention/concentration in the schizophrenia group.

Previous studies have shown that verbal memory impairment is more severe than visual memory impairment in patients with schizophrenia^3,27,28^. It has also been reported that memory impairment is more severe than impairments in general cognition, such as deficits in attention and vigilance, in patients with schizophrenia^2,3,29^. We replicated these findings in the current study. Relevant to the MRI volumes including the hippocampal and NA volumes, we replicated the results of Okada et al.^17^. Comparison of FreeSurfer estimates of NA volumes with those from manual tracings suggests that they are valid and comparable in reliability to those obtained in a previous study^30^. Our findings of significant correlations between memory indices and hippocampal volume in schizophrenia are consistent with previous studies^11,12,14^.

Previous studies showed negative correlation between memory performance and doses of antipsychotics or antiparkinsonian drugs in patients with schizophrenia^31,32^. Our results were consistent with these results. Relevant to the MRI indices, we also replicated previous studies, which showed no significant correlation between the hippocampal volumes and antipsychotics^25,33^. Although we could not find previous studies reporting on the association between the regional brain volumes and antiparkinsonian drugs, our results showed significant correlation between hippocampal/NA volumes and antiparkinsonian drugs. However, our main results remained almost the same even after adjusting for these medications.

The main novel finding of this study was the significant correlation between the verbal immediate recall/delayed recall index and NA volume in the schizophrenia group. There may be two explanations for the correlation. First, motivation supported by NA may be important for cognitive performance in schizophrenia. The function of the NA in the reward system is well known^34^, whereas its role in memory performance is not clear. In the current study, association between the NA volume and memory performance was not significantly stronger than the association between the NA volume and general cognitive performance in schizophrenia, because the general cognitive index was correlated with right NA volume at trend level (*p* < 0.10/42; Table 3). This finding is consistent with the first explanation. Green et al.^35^. assessed the perceptions, abilities, beliefs, motivations, and functional outcomes of patients with schizophrenia, and concluded that both ability and motivation were required for the community functioning of persons with schizophrenia. Fervaha et al.^36^. found an association between intrinsic motivation and cognitive test performance in patients with schizophrenia, and suggested that test performance was not purely a measure of ability. Motivational drive by the NA may be a major rate-limiting factor in the performance of cognitive-load-demanding tasks, such as verbal immediate/delayed recall in patients with schizophrenia.

The second possible interpretation is that the NA is important for memory per se. In this study, verbal immediate recall/delayed recall was significantly correlated with NA volume, when adjusting for attention/concentration in the schizophrenia group. This finding is consistent with the explanation. It is already known that the NA has learning and memory functions in rodents^37,38^. α-amino-3-hydroxy-5-methylisoxa-zole-4-propionic acid and N-methyl-D-aspartic acid receptors within the NA mediate different information-processing steps in mice^39,40^. The NA neuroanatomically receives convergent inputs from the hippocampus in mice^41,42^. Mice with mutant type III Neuregulin I, a schizophrenia susceptibility gene, have disrupted activity in the hippocampal–NA circuit^43^. Functional MRI has been used to show that there is connectivity between the hippocampus and the NA in normal human subjects^44^. We suggest that the NA has a strong connection with the hippocampus, and that volume reduction of the NA might affect memory impairment along with hippocampal volume reduction in patients with schizophrenia.

In HCS, several previous studies reported positive correlation between hippocampal volume and memory performance^45–48^, while Foster et al.^49^ reported negative correlation between them. Therefore, previous studies do not show consistent results of correlation between hippocampal volume and memory performance in HCS. Barnes et al.^50^ concluded that for region of interests (ROIs) analysis age and total intracranial volume (TIV) are strongly associated with volumes, and when considering ROI analysis, simply dividing by TIV is unlikely to be appropriate. In the current study, we normalized hippocampal volumes with age, sex, ICV, and MRI machine in the larger samples. Therefore, we considered that the current study might show more accurate results. In HCS, any relationship between hippocampus/NA volumes and memory performance might be obscured by the fact that these volumes are relatively retained within a restricted range (Supplementary Figure 8, 9).

Our study has some limitations. First, our study design was unable to disentangle *how* the NA contributes to memory impairments in schizophrenia. Future functional neuroimaging studies will be necessary to clarify this issue. Second, we need to clarify whether the NA’s contribution to memory performance deficits is specific to schizophrenia or whether it is also common to other neuropsychiatric disorders, such as major depression. Third, we were unable to find whether correlations with NA volume were specific to memory or whether they affected general cognitive performance. Since the Attention/Concentration index consists of the Mental Control, Digit Span, and Visual Memory Span subscales, general memory performance including working memory might contribute to performance in this measure to some extent. Fourth, a medication effect was not completely ruled out in our study. Fifth, we did not directly assess the motivational factor. Therefore, the accuracy of evaluating the association between motivation and the NA was limited.

In conclusion, the present investigation with a large sample size did not only replicate findings of an association between memory and hippocampal volume, but also found that NA volume is associated with memory performance in schizophrenia. These findings may prioritize clarification of how NA dysfunction affects memory impairment and may be important in the next steps toward developing biological and psychological strategies for improving cognitive and social function through ameliorating impairment of memory performance via stimulating NA in individuals with schizophrenia.

## Electronic supplementary material

Supplementary Text

Supplementary Tables

Supplementary Figure Legends

Supplementary Figure 1

Supplementary Figure 2

Supplementary Figure 3

Supplementary Figure 4

Supplementary Figure 5

Supplementary Figure 6

Supplementary Figure 7

Supplementary Figure 8

Supplementary Figure 9
