# Supplementary material for: Subcortical association with memory performance in schizophrenia: a structural magnetic resonance imaging study
Source: Transl Psychiatry. 2018 Jan 10;8:20. doi: 10.1038/s41398-017-0069-3 (PMC5802568; doi:10.1038/s41398-017-0069-3)
Supplement: Supplementary file 1 — Supplementary Text [file 41398_2017_69_MOESM1_ESM.docx]

**Manual tracing analysis of nucleus accumbens**

*Method*

Confirmation purpose of segmentation of nucleus accubens with FreeSurfer, we performed manual tracing of nucleus accumbens by three blinded raters on 40 randomly selected subjects; 10 with schizophrenia in Osaka A, 10 with schizophrenia in Osaka B, 10 with HCS in Osaka A, and 10 with HCS in Osaka B, respectively. The T1-weighted images were manually aligned parallel to the anterior and posterior commissure (AC-PC) line, and origin of the images was set at AC. The images aligned with AC-PC were then corrected for intensity inhomogeneity using Statistical Parametric Mapping (SPM) 12 (http://www.fil.ion.ucl.ac.uk/spm/), and resampled into 1mm isotropic voxels. We employed 3D-Slicer, an image-editing tool (http://www.slicer.org) for manual tracing. To describe nucleus accumbens, we put criteria as below reference to previous studies ^1, 2^. The first anterior slice of the nucleus accumbens was defined to start four slices after the putamen was first seen, while the last slice was defined to be two slices anterior to the anterior commissure in coronal plane. The nucleus accumbens was superiorly bordered by a line, which was drawn from the most inferiolateral voxel of the lateral ventricle to the most inferiomedial voxel of the putamen before the caudate and putamen were connected inferiorly. Once the caudate and putamen were visualized as an integrated structure, the superior border of the accumbens was marked by a line connecting the most inferiolateral voxel of the lateral ventricle to the most inferiolateral point of the internal capsule; while its lateral border was defined by a vertical line drawn from the internal capsule’s most inferiolateral voxel. In slices where branched anterior commissure was visible, the most lateral point of the branched anterior commissure was used as the starting point of the vertical line separating the putamen and the nucleus accumbens. We measured volumes by summing all the voxels within specified region of interests. Three-dimensional surface model of nucleus accumbens were constructed for confirmation purpose, using the region of interests (ROI) images aligned in MNI template space. As the ROI images of all the subjects were spatially aligned on a standard template, the mean of each voxel value represents the probability of the existence of the nucleus accumbens at the position. We used the boundary cutoff of 30% (0.3) for this probability to construct the surface model.

Intraclass correlation coefficients [ICC (3,1) with a two-way mixed model (consistency)] were used to evaluate inter-rater reliability for the manual tracing of nucleus accumbens volumes among three blinded raters, since the data showed a normal distribution. For comparison between nucleus accumbens volumes segmented by manual tracing and FreeSurfer, Spearman’s correlation coefficients were employed, because the data did not show a normal distribution. When comparing with FreeSurfer, we employed the voxels of nucleus accumbens traced by more than two raters among three blinded raters as the region representing of manual tracing. Dice’s coefficients were used to evaluate spatial overlap of manual tracing among three blinded raters. Dice’s coefficients were also employed to compare spatial overlap of nucleus accumbens voxels segmented by manual tracing with the voxels segmented by FreeSurfer.

*Results*

Inter-rater reliability scores for manual tracing of nucleus accumbens volumes among three blinded raters were shown in **Supplementary Table 1**; the ICCs were from 0.603 to 0.812 (0.41 ≤ICC ≤0.60 is considered moderate; 0.61 ≤ICC ≤0.8 is considered substantial ^3^). Correlation coefficients between nucleus accumbens volumes segmented by manual tracing and the volumes segmented by FreeSurfer were shown in **Supplementary Table 2**; the correlation coefficients were from 0.503 to 0.842. Dice’s coefficients of nucleus accumbens with manual tracing among three blinded raters for evaluating spatial overlap were shown in **Supplementary Table 3**; the Dice’s coefficients were from 0.659 to 0.756. Dice’s coefficients between nucleus accumbens volumes segmented by manual tracing and the volumes segmented by FreeSurfer were shown in **Supplementary Table 4**; the Dice’s coefficients were from 0.527 to 0.665. Three-dimensional surface models of nucleus accumbens were shown in **Supplementary Figure 1**.

**References**

1. Langen M, Durston S, Staal WG, Palmen SJ, van Engeland H. Caudate nucleus is enlarged in high-functioning medication-naive subjects with autism. *Biol Psychiatry* 2007; **62**(3)**:** 262-266.

2. Loh WY, Connelly A, Cheong JL, Spittle AJ, Chen J, Adamson C *et al.* A New MRI-Based Pediatric Subcortical Segmentation Technique (PSST). *Neuroinformatics* 2016; **14**(1)**:** 69-81.

3. Landis JR, Koch GG. The measurement of observer agreement for categorical data. *Biometrics* 1977; **33**(1)**:** 159-174.
