# Supplementary material for: Subcortical association with memory performance in schizophrenia: a structural magnetic resonance imaging study
Source: Transl Psychiatry. 2018 Jan 10;8:20. doi: 10.1038/s41398-017-0069-3 (PMC5802568; doi:10.1038/s41398-017-0069-3)
Supplement: Supplementary file 2 — Supplementary Tables [file 41398_2017_69_MOESM2_ESM.docx]

**Supplementary Table 1** Inter-rater reliability scores for manual tracing of nucleus accumbens volumes among three blinded raters in each MRI machine in patients with schizophrenia and healthy comparison subjects.

|  | Intraclass Correlation Coefficient | |
| --- | --- | --- |
|  | L Accumbens | R Accumbens |
| Osaka A, SZ | 0.603 | 0.783 |
| Osaka B, SZ | 0.639 | 0.791 |
| Osaka A, HCS | 0.755 | 0.812 |
| Osaka B, HCS | 0.742 | 0.629 |

Abbreviation: MRI, magnetic resonance imaging; SZ, schizophrenia; HCS, healthy comparison subjects; L, left; R, right.

**Supplementary Table 2** Correlation coefficients between nucleus accumbens volumes segmented by manual tracing and nucleus accumbens volumes segmented by FreeSurfer in each MRI machine in patients with schizophrenia and healthy comparison subjects.

|  |  |  | FreeSurfer | |
| --- | --- | --- | --- | --- |
|  |  |  | L Accumbens | R Accumbens |
|  |  |  | *r* | *r* |
| Manual tracing | |  |  |  |
|  | Osaka A, SZ | L Accumbens | 0.842 |  |
|  |  | R Accumbens |  | 0.842 |
|  | Osaka B, SZ | L Accumbens | 0.685 |  |
|  |  | R Accumbens |  | 0.794 |
|  | Osaka A, HCS | L Accumbens | 0.552 |  |
|  |  | R Accumbens |  | 0.818 |
|  | Osaka B, HCS | L Accumbens | 0.644 |  |
|  |  | R Accumbens |  | 0.503 |

Legends: The voxels of nucleus accumbens volumes segmented by manual tracing were decided with tracing by more than two raters among three blinded raters.

Abbreviation: MRI, magnetic resonance imaging; L, left; R, right; *r*, Spearman’s correlation coefficient; SZ, schizophrenia; HCS, healthy comparison subjects.

**Supplementary Table 3** Dice’s coefficients of nucleus accumbens with manual tracing among three blinded raters A, B, and C for evaluating spatial overlap in each MRI machine in patients with schizophrenia and healthy comparison subjects.

|  |  | Dice’s coefficients | | | | | | | |
| --- | --- | --- | --- | --- | --- | --- | --- | --- | --- |
|  |  | Osaka A, SZ | | Osaka B, SZ | | Osaka A, HC | | Osaka A, HC | |
|  |  | Rater A | Rater B | Rater A | Rater B | Rater A | Rater B | Rater A | Rater B |
| L Accumbens | |  |  |  |  |  |  |  |  |
|  | Rater B | 0.682 |  | 0.707 |  | 0.691 |  | 0.676 |  |
|  | Rater C | 0.659 | 0.676 | 0.732 | 0.723 | 0.669 | 0.692 | 0.736 | 0.700 |
| R Accumbens | |  |  |  |  |  |  |  |  |
|  | Rater B | 0.675 |  | 0.721 |  | 0.701 |  | 0.730 |  |
|  | Rater C | 0.697 | 0.677 | 0.756 | 0.743 | 0.727 | 0.711 | 0.747 | 0.682 |

Abbreviation: MRI, magnetic resonance imaging; L, left; R, right; SZ, schizophrenia; HCS, healthy comparison subjects.

**Supplementary Table 4** Dice’s coefficients between nucleus accumbens voxels segmented by manual tracing and nucleus accumbens voxels segmented by FreeSurfer for evaluating spatial overlap in each MRI machine in patients with schizophrenia and healthy comparison subjects.

|  | Dice’s coefficients | |
| --- | --- | --- |
|  | L Accumbens | R Accumbens |
| Osaka A, SZ | 0.616 | 0.543 |
| Osaka B, SZ | 0.653 | 0.545 |
| Osaka A, HCS | 0.638 | 0.539 |
| Osaka B, HCS | 0.665 | 0.527 |

Legends: The voxels of nucleus accumbens segmented by manual tracing were decided with tracing by more than two raters among three blinded raters.

Abbreviation: MRI, magnetic resonance imaging; L, left; R, right; SZ, schizophrenia; HCS, healthy comparison subjects.

**Supplementary Table 5** Partial correlation coefficients between verbal immediate recall/delayed recall and nucleus accumbens volume after adjusting for Attention/Concentration in patients with schizophrenia.

|  |  | Accumbens | | | | |
| --- | --- | --- | --- | --- | --- | --- |
|  |  | L | |  | R | |
|  |  | Original | Attention/Concentration |  | Original | Attention/Concentration |
| Verbal Memory | *r* | 0.244 | 0.229 |  | 0.246 | 0.165 |
|  | *p* | 1.17×10^-3^ | 2.4×10^-3^ |  | 1.1×10^-3^ | 3.0×10^-2^ |
| Delayed Recall | *r* | 0.255 | 0.244 |  | 0.27 | 0.185 |
|  | *p* | 7.0×10^-4^ | 1.2×10^-3^ |  | 3.1×10^-4^ | 1.5×10^-2^ |

Legend: First column shows original correlation coefficients between verbal immediate recall/delayed recall and nucleus accumbens in patients with schizophrenia. Second column shows partial correlation coefficients between verbal immediate recall/delayed recall and nucleus accumbens volume in patients with schizophrenia adjusted for Attention/Concentration. A *p*-value of < 1.19 × 10^-3^ (0.05/42) was considered statistically significant for the original correlation coefficients. A *p*-value <0.05 was considered statistically significant for partial correlation coefficients adjusted for Attention/Concentration. Underline indicates *p* < 0.05.

Abbreviation: *r*, Pearson correlation coefficient; L, left; R, right.

**Supplementary Table 6** Partial correlation coefficients between verbal immediate recall/delayed recall and hippocampus/nucleus accumbens volume after adjusting hippocampal volume in patients with schizophrenia.

|  |  | Accumbens | | | | |
| --- | --- | --- | --- | --- | --- | --- |
|  |  | L | |  | R | |
|  |  | Original | Partial out of hippocampal volume |  | Original | Partial out of hippocampal volume |
| Verbal Memory | *r* | 0.244 | 0.153 |  | 0.246 | 0.143 |
|  | *p* | 1.17×10^-3^ | 4.4×10^-2^ |  | 1.1×10^-3^ | 6.1×10^-2^ |
| Delayed Recall | *r* | 0.255 | 0.161 |  | 0.270 | 0.167 |
|  | *p* | 7.0×10^-4^ | 3.4×10^-2^ |  | 3.1×10^-4^ | 2.8×10^-2^ |

Legend: First column shows original correlation coefficients between verbal immediate recall/delayed recall and hippocampus/nucleus accumbens volume in patients with schizophrenia. Second column shows partial correlation coefficients between verbal immediate recall/delayed recall and hippocampus/nucleus accumbens volume in patients with schizophrenia adjusted for hippocampal volume. A *p*-value of < 1.19 × 10^-3^ (0.05/42) was considered statistically significant for the original correlation coefficients. A *p*-value <0.05 was considered statistically significant for partial correlation coefficients adjusted for hippocampal volume.

Abbreviation: *r*, Pearson correlation coefficient; L, left; R, right.

**Supplementary Table 7** Partial correlation coefficients between verbal immediate recall/delayed recall and hippocampus/nucleus accumbens volume after adjusting premorbid IQ in patients with schizophrenia.

|  |  | Hippocampus | | | | |  | Accumbens | | | | |
| --- | --- | --- | --- | --- | --- | --- | --- | --- | --- | --- | --- | --- |
|  |  | L | |  | R | |  | L | |  | R | |
|  |  | Original | Premorbid IQ |  | Original | Premorbid IQ |  | Original | Premorbid IQ |  | Original | Premorbid IQ |
| Verbal Memory | *r* | 0.339 | 0.225 |  | 0.281 | 0.167 |  | 0.244 | 0.228 |  | 0.246 | 0.189 |
|  | *p* | 4.6×10^-6^ | 3.3×10^-3^ |  | 1.8×10^-4^ | 3.0×10^-2^ |  | 1.17×10^-3^ | 2.9×10^-3^ |  | 1.1×10^-3^ | 1.4×10^-2^ |
| Delayed Recall | *r* | 0.333 | 0.221 |  | 0.310 | 0.214 |  | 0.255 | 0.233 |  | 0.270 | 0.217 |
|  | *p* | 6.9×10^-6^ | 3.9×10^-3^ |  | 3.2×10^-5^ | 5.2×10^-3^ |  | 7.0×10^-4^ | 2.3×10^-3^ |  | 3.1×10^-4^ | 4.7×10^-3^ |

Legend: First column shows original correlation coefficients between verbal immediate recall/delayed recall and hippocampus/nucleus accumbens in patients with schizophrenia. Second column shows partial correlation coefficients between verbal immediate recall/delayed recall and hippocampus/nucleus accumbens volume in patients with schizophrenia adjusted for premorbid IQ. A *p*-value of < 1.19 × 10^-3^ (0.05/42) was considered statistically significant for the original correlation coefficients. A *p*-value <0.05 was considered statistically significant for partial correlation coefficients adjusted for premorbid IQ.

Abbreviation: IQ, intelligence quotient; *r*, Pearson correlation coefficient; L, left; R, right.

**Supplementary Table 8** Partial correlation coefficients between verbal immediate recall/delayed recall and hippocampus/nucleus accumbens volume after adjusting the duration of illness (years) in patients with schizophrenia.

|  |  | Hippocampus | | | | |  | Accumbens | | | | |
| --- | --- | --- | --- | --- | --- | --- | --- | --- | --- | --- | --- | --- |
|  |  | L | |  | R | |  | L | |  | R | |
|  |  | Original | Duration of illness |  | Original | Duration of illness |  | Original | Duration of illness |  | Original | Duration of illness |
| Verbal Memory | *r* | 0.339 | 0.342 |  | 0.281 | 0.277 |  | 0.244 | 0.246 |  | 0.246 | 0.242 |
|  | *p* | 4.6×10^-6^ | 4.3×10^-6^ |  | 1.8×10^-4^ | 2.3×10^-4^ |  | 1.17×10^-3^ | 1.1×10^-3^ |  | 1.1×10^-3^ | 1.3×10^-3^ |
| Delayed Recall | *r* | 0.333 | 0.337 |  | 0.310 | 0.305 |  | 0.255 | 0.258 |  | 0.270 | 0.267 |
|  | *p* | 6.9×10^-6^ | 5.7×10^-6^ |  | 3.2×10^-5^ | 4.4×10^-5^ |  | 7.0×10^-4^ | 6.0×10^-4^ |  | 3.1×10^-4^ | 3.9×10^-4^ |

Legend: First column shows original correlation coefficients between verbal immediate recall/delayed recall and nucleus accumbens in patients with schizophrenia. Second column shows partial correlation coefficients between verbal immediate recall/delayed recall and hippocampus/nucleus accumbens volume in patients with schizophrenia adjusted for the duration of illness (years). A *p*-value of < 1.19 × 10^-3^ (0.05/42) was considered statistically significant for the original correlation coefficients. A *p*-value <0.05 was considered statistically significant for partial correlation coefficients adjusted for the duration of illness.

Abbreviation: IQ, intelligence quotient; *r*, Pearson correlation coefficient; L, left; R, right.

**Supplementary Table 9** Partial correlation coefficients between verbal immediate recall/delayed recall and hippocampus/nucleus accumbens volume in patients with schizophrenia adjusted for time interval between the WMS-R measurements and the MRI scanning, and correlation coefficients between verbal immediate recall/delayed recall and hippocampus/nucleus accumbens volume only in 147 patients with schizophrenia within three months time interval between the WMS-R measurements and the MRI scanning.

|  |  | Hippocampus | | | | | | |  | Accumbens | | | | | | |
| --- | --- | --- | --- | --- | --- | --- | --- | --- | --- | --- | --- | --- | --- | --- | --- | --- |
|  |  | L | | |  | R | | |  | L | | |  | R | | |
|  |  | Original | Partial correlation | Within 3 months |  | Original | Partial correlation | Within 3 months |  | Original | Partial correlation | Within 3 months |  | Original | Partial correlation | Within 3 months |
| Verbal Memory | *r* | 0.339 | 0.335 | 0.328 |  | 0.281 | 0.276 | 0.271 |  | 0.244 | 0.237 | 0.251 |  | 0.246 | 0.238 | 0.238 |
|  | *p* | 4.6×10^-6^ | 6.7×10^-6^ | 4.9×10^-5^ |  | 1.8×10^-4^ | 2.4×10^-4^ | 9.0×10^-4^ |  | 1.17×10^-3^ | 1.7×10^-3^ | 2.1×10^-3^ |  | 1.1×10^-3^ | 1.6×10^-3^ | 3.6×10^-3^ |
| Delayed Recall | *r* | 0.333 | 0.332 | 0.332 |  | 0.310 | 0.308 | 0.319 |  | 0.255 | 0.253 | 0.264 |  | 0.270 | 0.269 | 0.270 |
|  | *p* | 6.9×10^-6^ | 8.1×10^-6^ | 4.0×10^-5^ |  | 3.2×10^-5^ | 3.7×10^-5^ | 8.1×10^-5^ |  | 7.0×10^-4^ | 7.8×10^-4^ | 1.2×10^-3^ |  | 3.1×10^-4^ | 3.5×10^-4^ | 9.6×10^-4^ |

Legend: First column shows original correlation coefficients between verbal immediate recall/delayed recall and hippocampus/nucleus accumbens volume in all 174 patients with schizophrenia. Second column shows partial correlation coefficients between verbal immediate recall/delayed recall and hippocampus/nucleus accumbens volume in patients with schizophrenia adjusted for time interval between the WMS-R measurements and the MRI scanning. All results were significant. Third column shows correlation coefficients between verbal immediate recall/delayed recall and hippocampus/ nucleus accumbens volume only in 147 patients with schizophrenia within three months time interval between the WMS-R measurements and the MRI scanning. A *p*-value of < 1.19 × 10^-3^ (0.05/42) was considered statistically significant for the original correlation coefficients. A *p*-value <0.05 was considered statistically significant for partial correlation coefficients adjusted for time interval, and correlation coefficients only in patients with schizophrenia within three months time interval.

Abbreviations: WMS-R, Wechsler Memory Scale-Revised; MRI, magnetic resonance imaging; L, left; R; right; *r*, Pearson correlation coefficient.

**Supplementary Table 10** Correlation analyses on our main findings for each MRI machine: Osaka A and Osaka B, in patients with schizophrenia.

|  |  |  | Hippocampus | | Accumbens | |
| --- | --- | --- | --- | --- | --- | --- |
|  |  |  | L | R | L | R |
| Osaka A | |  |  |  |  |  |
|  | Verbal Memory | *r* | 0.323 | 0.184 | 0.33 | 0.296 |
|  |  | *p* | 6.9×10^-4^ | 5.8×10^-2^ | 5.1×10^-4^ | 2.0×10^-3^ |
|  | Delayed Recall | *r* | 0.277 | 0.183 | 0.259 | 0.278 |
|  |  | *p* | 3.8×10^-3^ | 5.9×10^-2^ | 7.1×10^-3^ | 3.8×10^-3^ |
| Osaka B | |  |  |  |  |  |
|  | Verbal Memory | *r* | 0.36 | 0.392 | 0.134 | 0.19 |
|  |  | *p* | 2.7×10^-3^ | 1.0×10^-3^ | 0.28 | 0.12 |
|  | Delayed Recall | *r* | 0.416 | 0.48 | 0.253 | 0.272 |
|  |  | *p* | 4.6×10^-4^ | 4.0×10^-5^ | 3.9×10^-2^ | 2.6×10^-2^ |

Legend: We used corrected regional volumes, which regressed the effects of age, sex, and ICV. A *p*-value <0.05 was considered statistically significant. Underline indicates *p* < 0.05.

Abbreviation: ICV, intracranial volume; L, left; R, right.

**Supplementary Table 11** Correlation between medication and verbal immediate recall, delayed recall, hippocampus volume, and nucleus accumbens volume in patients with schizophrenia.

|  |  |  | Chlorpromazine equivalent (mg/day) | Diazepam equivalent (mg/day) | Biperiden equivalent (mg/day) |
| --- | --- | --- | --- | --- | --- |
| Verbal Memory |  | *r* | -0.173 | -0.094 | -0.087 |
|  |  | *p* | 2.3×10^-2^ | 0.22 | 0.26 |
| Delayed Recall |  | *r* | -0.261 | -0.097 | -0.154 |
|  |  | *p* | 5.1×10^-4^ | 0.20 | 4.2×10^-2^ |
| Hippocampus | L | *r* | -0.083 | -0.054 | -0.219 |
|  |  | *p* | 0.274 | 0.478 | 3.7×10^-3^ |
|  | R | *r* | -0.112 | -0.085 | -0.194 |
|  |  | *p* | 0.14 | 0.26 | 1.0×10^-2^ |
| Accumbens | L | *r* | -0.007 | 0.037 | -0.093 |
|  |  | *p* | 0.923 | 0.631 | 0.220 |
|  | R | *r* | -0.091 | -0.007 | -0.152 |
|  |  | *p* | 0.23 | 0.93 | 4.6×10^-2^ |

Legend: Underline indicates *p* < 0.05.

Abbreviation: *r*, Pearson correlation coefficient; L, left; R, right.

**Supplementary Table 12** Partial correlation coefficients between verbal immediate recall/delayed recall and hippocampus/nucleus accumbens volume in patients with schizophrenia adjusted for medication.

|  |  | Hippocampus | | | | | | |  | Accumbens | | | | | | |
| --- | --- | --- | --- | --- | --- | --- | --- | --- | --- | --- | --- | --- | --- | --- | --- | --- |
|  |  | L | | |  | R | | |  | L | | |  | R | | |
|  |  | Original | CPZ equivalent | Biperiden equivalent |  | Original | CPZ equivalent | Biperiden equivalent |  | Original | CPZ equivalent | Biperiden equivalent |  | Original | CPZ equivalent | Biperiden equivalent |
| Verbal Memory | *r* | 0.339 | 0.331 | 0.330 |  | 0.281 | 0.267 | 0.270 |  | 0.244 | 0.247 |  |  | 0.246 | 0.234 | 0.236 |
|  | *p* | 4.6×10^-6^ | 8.6×10^-6^ | 9.5×10^-6^ |  | 1.8×10^-4^ | 3.8×10^-4^ | 3.3×10^-4^ |  | 1.17×10^-3^ | 1.1×10^-3^ |  |  | 1.1×10^-3^ | 1.9×10^-3^ | 1.8×10^-3^ |
| Delayed Recall | *r* | 0.333 | 0.324 | 0.311 |  | 0.310 | 0.292 | 0.289 |  | 0.255 | 0.262 | 0.244 |  | 0.270 | 0.256 | 0.253 |
|  | *p* | 6.9×10^-6^ | 1.4×10^-5^ | 3.2×10^-5^ |  | 3.2×10^-5^ | 9.5×10^-5^ | 1.2×10^-4^ |  | 7.0×10^-4^ | 5.0×10^-4^ | 1.2×10^-3^ |  | 3.1×10^-4^ | 6.6×10^-4^ | 7.9×10^-4^ |

Legend: First column shows original correlation coefficients between memory indices and MRI indices in patients with schizophrenia. Second and third columns show partial correlation coefficients between memory indices and MRI indices in patients with schizophrenia adjusted for chlorpromazine/biperiden equivalent. All results were significant. A *p*-value of < 1.19 × 10^-3^ (0.05/42) was considered statistically significant for the original correlation coefficients. A *p*-value <0.05 was considered statistically significant for partial correlation coefficients adjusted for chlorpromazine/biperiden equivalent.

Abbreviations: MRI, magnetic resonance imaging; CPZ, chlorpromazine; L, left; R; right; *r*, Pearson correlation coefficient.
