# Supplementary material for: Subcortical association with memory performance in schizophrenia: a structural magnetic resonance imaging study
Source: Transl Psychiatry. 2018 Jan 10;8:20. doi: 10.1038/s41398-017-0069-3 (PMC5802568; doi:10.1038/s41398-017-0069-3)
Supplement: Supplementary file 3 — Supplementary Figure Legends [file 41398_2017_69_MOESM3_ESM.docx]

**Supplementary figure legends**

**Supplementary Figure 1** Three-dimensional surface models of nucleus accumbens with manual trace and FreeSurfer in patients with schizophrenia and healthy comparison subjects in each MRI machine (Osaka A and Osaka B).

Legends: We employed the voxels of nucleus accumbens traced by more than two raters among three blinded raters as the region representing of manual tracing. As the ROI images of all the subjects were spatially aligned on a standard template, the mean of each voxel value represents the probability of the existence of the nucleus accumbens at the position. We used the boundary cutoff of 30% (0.3) for this probability to construct the surface model.

Abbreviations: MRI, magnetic resonance imaging; SZ, patients with schizophrenia; HCS, healthy comparison subjects; S, superior; L, left; R, right; I, inferior.

**Supplementary Figure 2** Histograms of the age distribution in the schizophrenia group and the healthy comparison subject group.

**Supplementary Figure 3** The correlation map among the regional brain volumes in all participants.

Abbreviations: L, left; R, right.

**Supplementary Figure 4** The correlation map among the regional brain volumes in schizophrenia group.

Abbreviations: L, left; R, right.

**Supplementary Figure 5** The correlation map among the regional brain volumes in healthy comparison subject group.

Abbreviations: L, left; R, right.

**Supplementary Figure 6** Correlation between the Verbal Memory (verbal immediate recall)/Delayed Recall Index and left (blue)/right (red) hippocampal volumes in patients with schizophrenia.

Abbreviations: L, left; R, right.

**Supplementary Figure 7** Correlation between the Verbal Memory (verbal immediate recall)/Delayed Recall Index and left (blue)/right (red) nucleus accumbens volumes in patients with schizophrenia.

Abbreviations: L, left; R, right.

**Supplementary Figure 8** Correlation between the Verbal Memory (verbal immediate recall)/Delayed Recall Index and left (blue)/right (red) hippocampal volumes in healthy comparison subjects.

Abbreviations: L, left; R, right.

**Supplementary Figure 9** Correlation between the Verbal Memory (verbal immediate recall)/Delayed Recall Index and left (blue)/right (red) nucleus accumbens volumes in healthy comparison subjects.

Abbreviations: L, left; R, right.
