# Supplementary figures and images for: Subcortical association with memory performance in schizophrenia: a structural magnetic resonance imaging study
Source: Transl Psychiatry. 2018 Jan 10;8:20. doi: 10.1038/s41398-017-0069-3 (PMC5802568; doi:10.1038/s41398-017-0069-3)

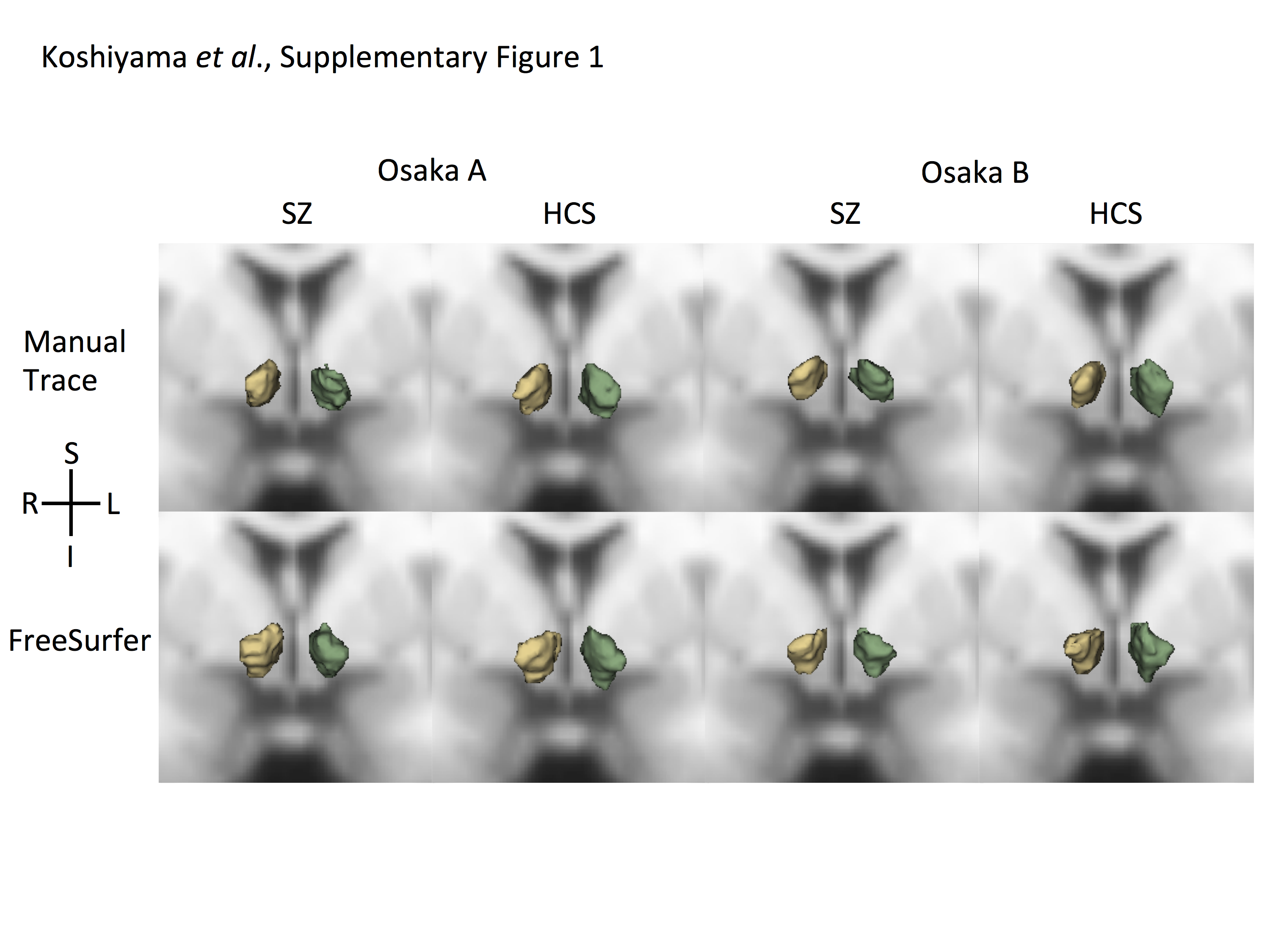

Supplement: Supplementary file 4 — Supplementary Figure 1 [file 41398_2017_69_MOESM4_ESM.tif]

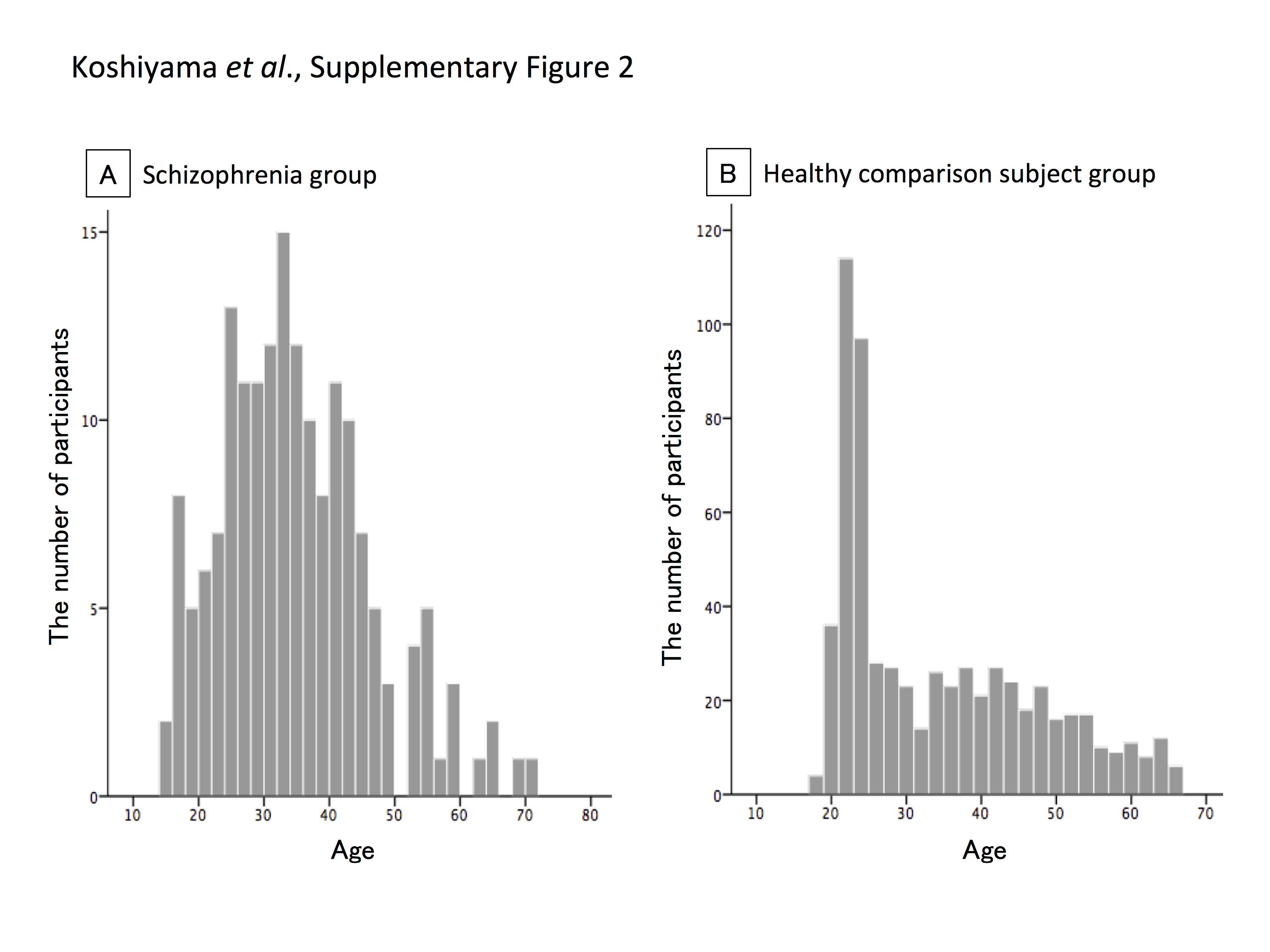

Supplement: Supplementary file 5 — Supplementary Figure 2 [file 41398_2017_69_MOESM5_ESM.tif]

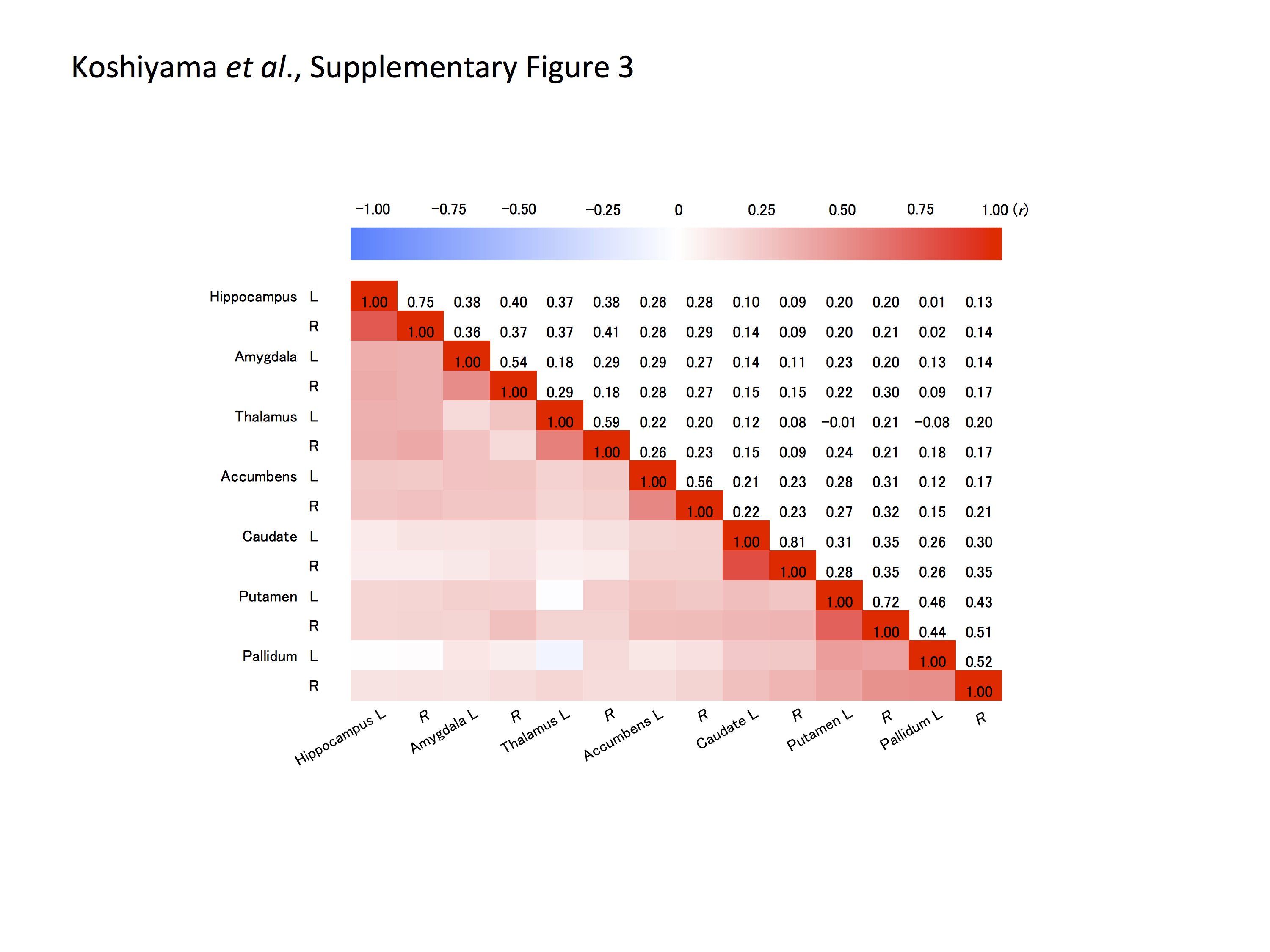

Supplement: Supplementary file 6 — Supplementary Figure 3 [file 41398_2017_69_MOESM6_ESM.tif]

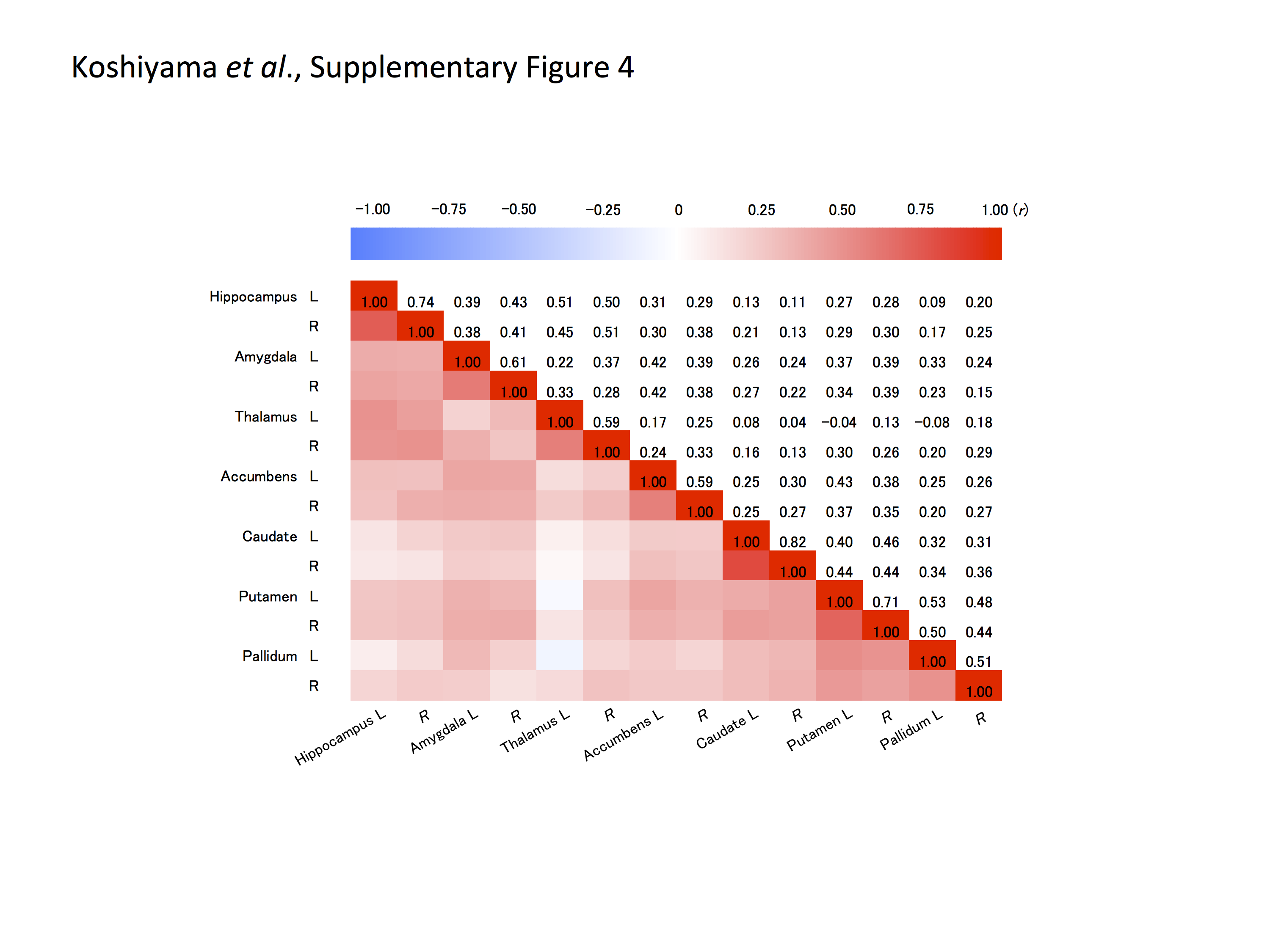

Supplement: Supplementary file 7 — Supplementary Figure 4 [file 41398_2017_69_MOESM7_ESM.tif]

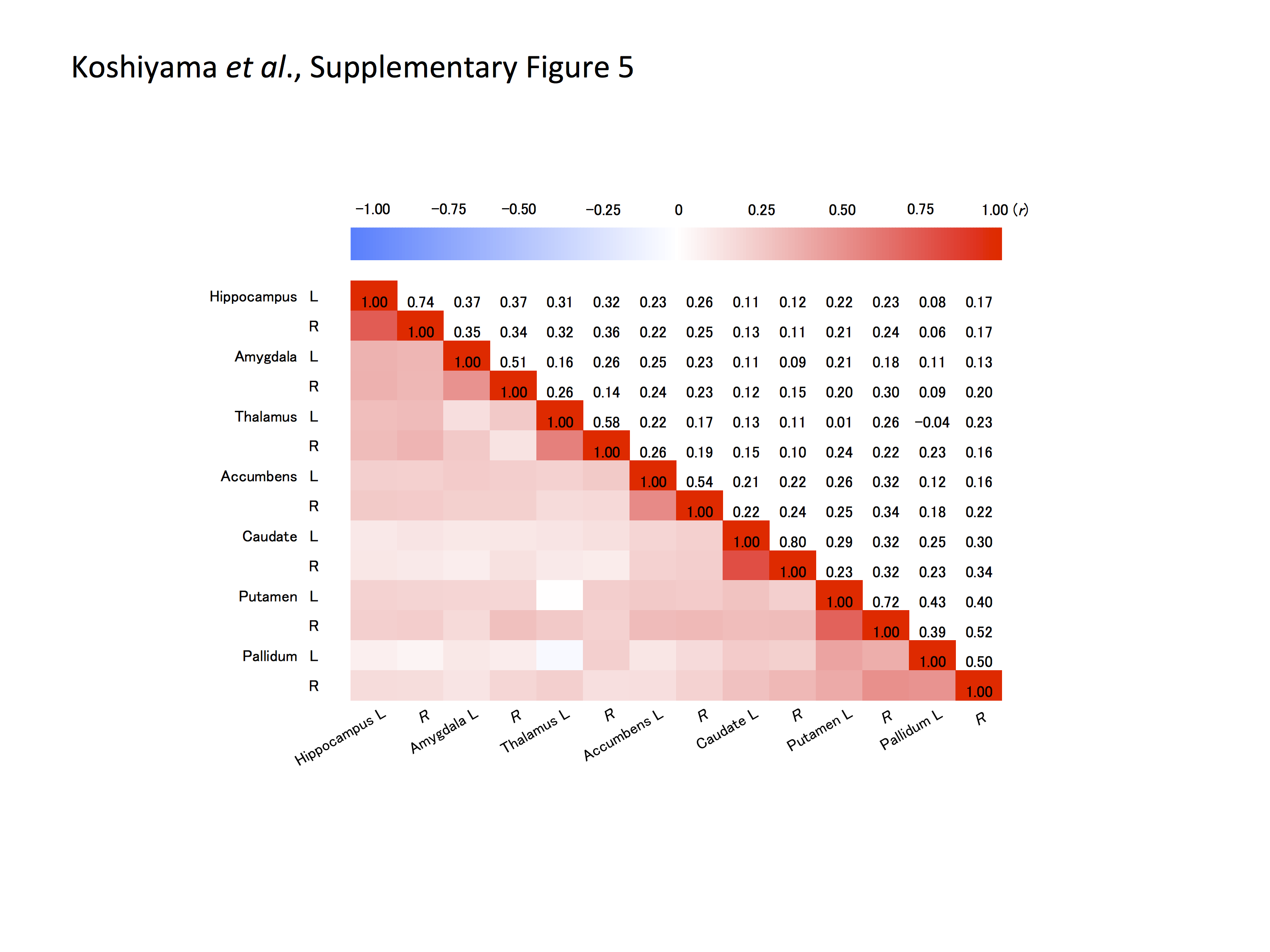

Supplement: Supplementary file 8 — Supplementary Figure 5 [file 41398_2017_69_MOESM8_ESM.tif]

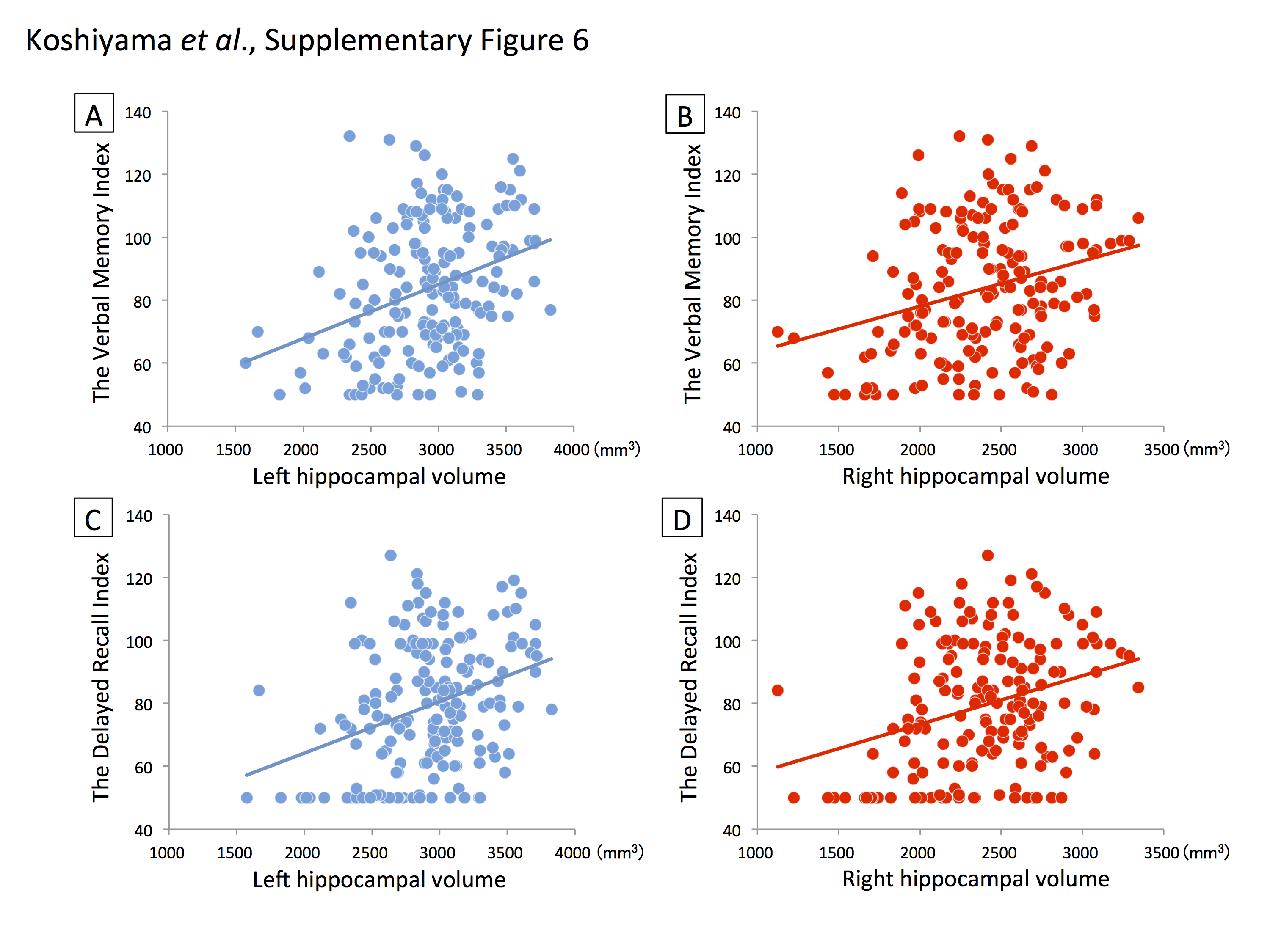

Supplement: Supplementary file 9 — Supplementary Figure 6 [file 41398_2017_69_MOESM9_ESM.tif]

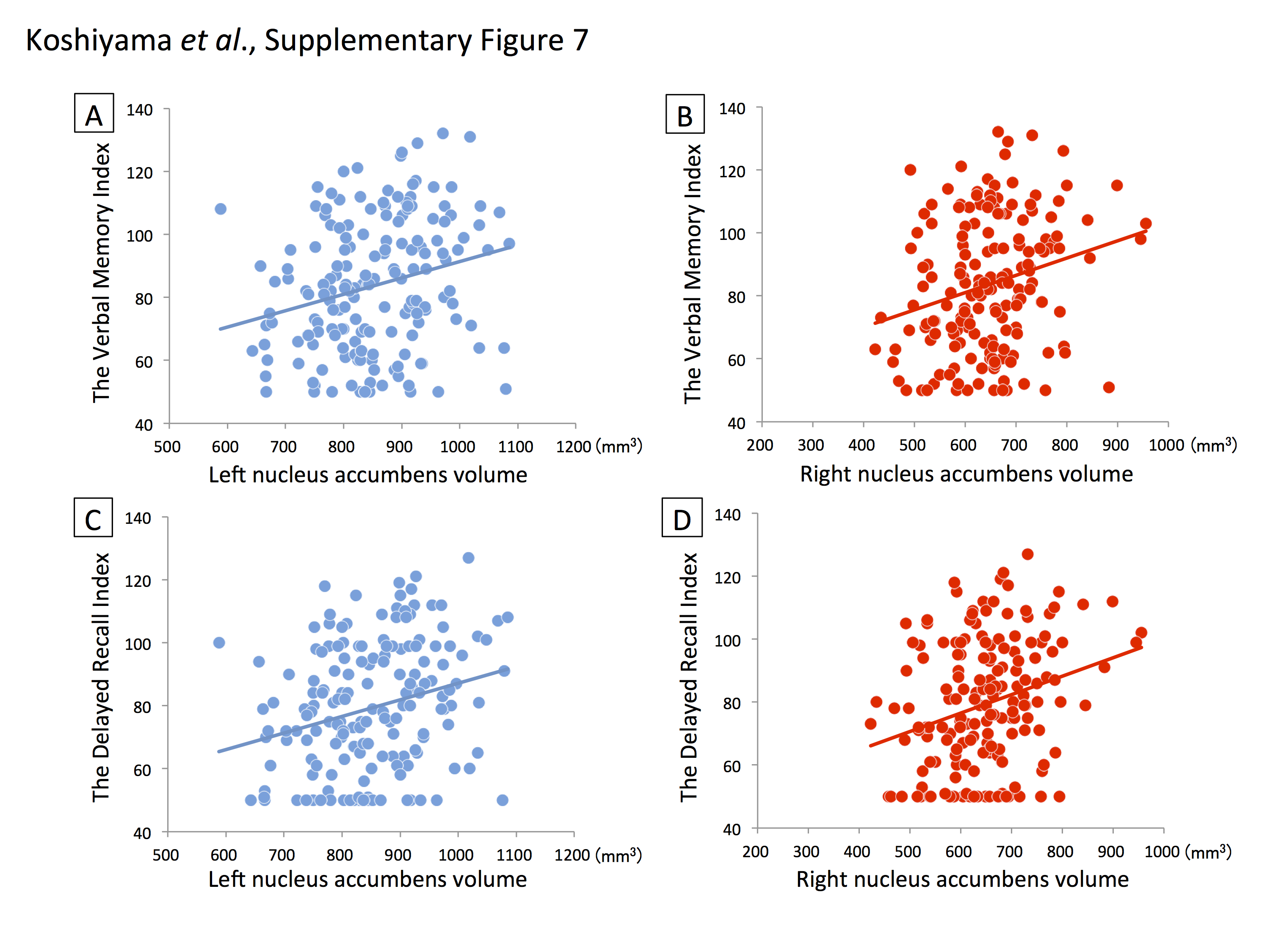

Supplement: Supplementary file 10 — Supplementary Figure 7 [file 41398_2017_69_MOESM10_ESM.tif]

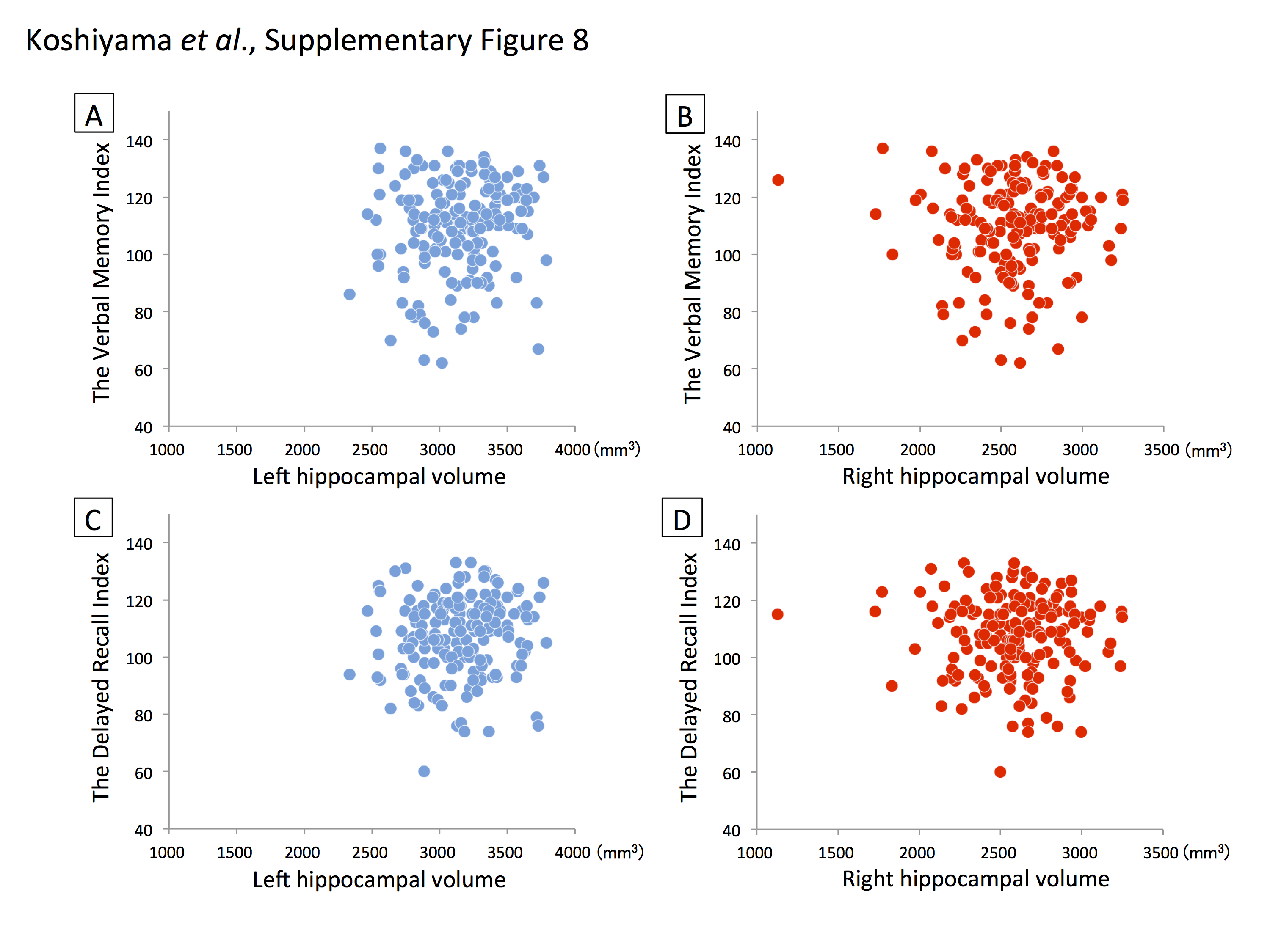

Supplement: Supplementary file 11 — Supplementary Figure 8 [file 41398_2017_69_MOESM11_ESM.tif]

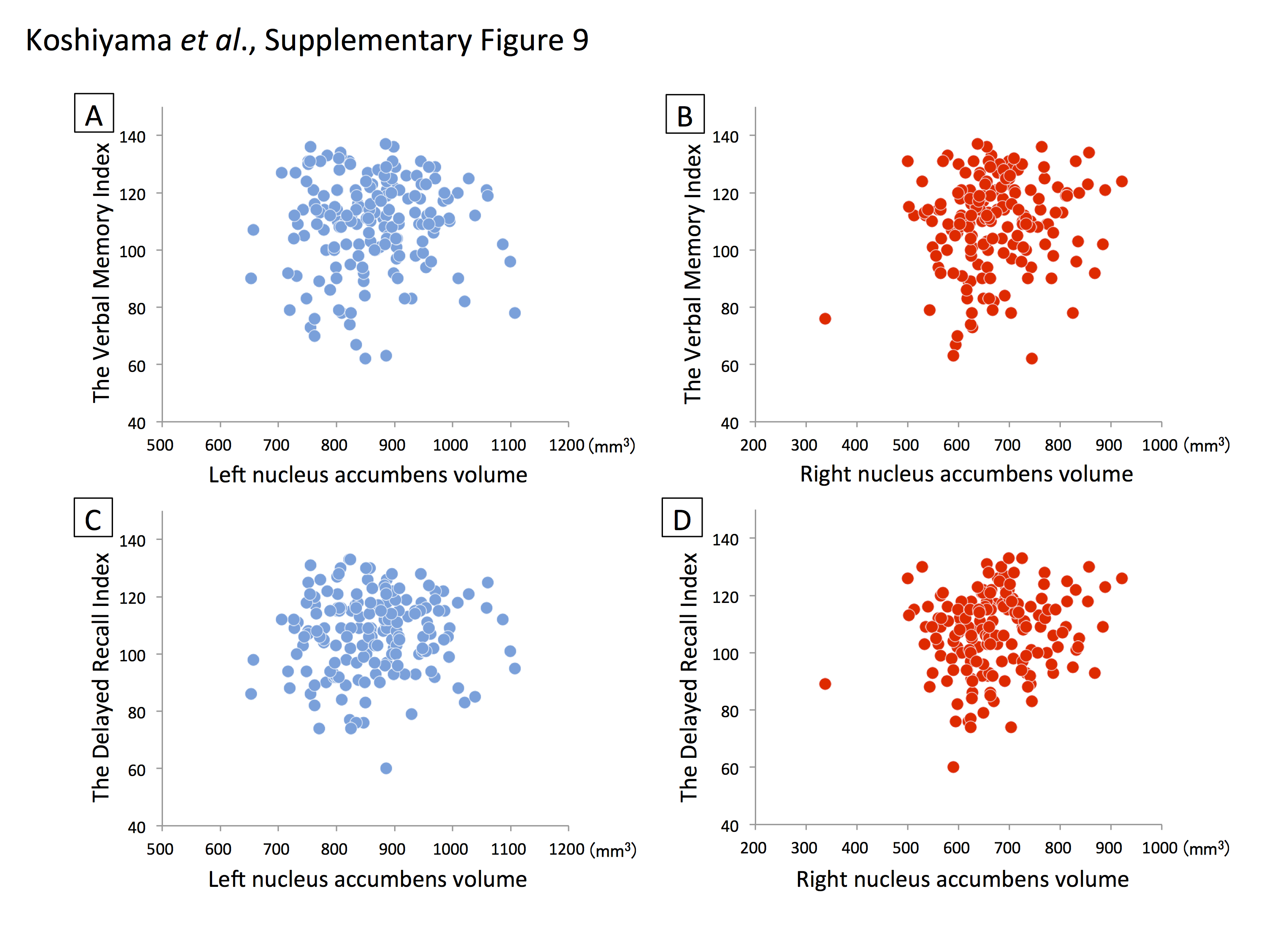

Supplement: Supplementary file 12 — Supplementary Figure 9 [file 41398_2017_69_MOESM12_ESM.tif]
